# ZNF512B associates with mitotic spindles, regulates metaphase exit, and is crucial for stem cell differentiation

**DOI:** 10.1093/nar/gkag802

**Published:** 2026-08-11

**Authors:** Lena W Paasche, Nadine Daus, Johannes J Groos, Felix Diegmüller, Carlotta Kreienbaum, Erekle Kobakhidze, Jie Lan, Jörg Leers, Stefanie Wunderlich, Marion Scheibe, Falk Butter, Annette Borchers, Joel P Mackay, Tim Marius Wunderlich, Sandra B Hake

**Affiliations:** Institute for Genetics, Justus Liebig University Giessen, 35392 Giessen, Germany; Institute for Genetics, Justus Liebig University Giessen, 35392 Giessen, Germany; Institute for Genetics, Justus Liebig University Giessen, 35392 Giessen, Germany; Institute for Genetics, Justus Liebig University Giessen, 35392 Giessen, Germany; Institute for Genetics, Justus Liebig University Giessen, 35392 Giessen, Germany; School of Life and Environmental Sciences, University of Sydney, New South Wales 2006, Australia; Institute for Genetics, Justus Liebig University Giessen, 35392 Giessen, Germany; Institute for Genetics, Justus Liebig University Giessen, 35392 Giessen, Germany; Department of Biology, Molecular Embryology, Marburg University, 35043 Marburg, Germany; Institute of Molecular Virology and Cell Biology, Friedrich-Loeffler-Institute, Federal Research Institute for Animal Health, 17493 Greifswald, Germany; Institute of Molecular Virology and Cell Biology, Friedrich-Loeffler-Institute, Federal Research Institute for Animal Health, 17493 Greifswald, Germany; Department of Biology, Molecular Embryology, Marburg University, 35043 Marburg, Germany; School of Life and Environmental Sciences, University of Sydney, New South Wales 2006, Australia; Institute for Genetics, Justus Liebig University Giessen, 35392 Giessen, Germany; Institute for Genetics, Justus Liebig University Giessen, 35392 Giessen, Germany

## Abstract

Zinc finger proteins are a large family of DNA-binding factors that play key roles in diverse cellular processes, including gene regulation, RNA metabolism, and cell cycle control. The zinc finger protein ZNF512B has recently been implicated in chromatin organization and transcriptional repression through its direct interaction with the nucleosome remodeling and deacetylase (NuRD) complex, its DNA-binding ability, and its association with the histone variant H2A.Z. Here, we uncover a previously unrecognized role for ZNF512B that is independent of both its zinc finger domains and NuRD association. We identify ZNF512B as a spindle-associated factor that regulates progression through mitosis, specifically controlling metaphase exit. ZNF512B’s internal domain interacts with chromosome segregation proteins and contains 25 repeat motifs, predicted to form a β-helix structure, which are required and sufficient for spindle interaction. Elevated ZNF512B levels result in a profound metaphase arrest that is ultimately lethal; a phenotype arising from the combined activity of its spindle-binding and chromatin-tethering functions. Conversely, ZNF512B depletion accelerates stem cell proliferation, impairs differentiation, and upregulates genes linked to cell cycle progression. Our findings position ZNF512B as a multifunctional protein that acts as a transcriptional repressor, a chromatin aggregator and a novel metaphase exit regulator through spindle fiber binding.

## Graphical Abstract

**Graphical Abstract:**
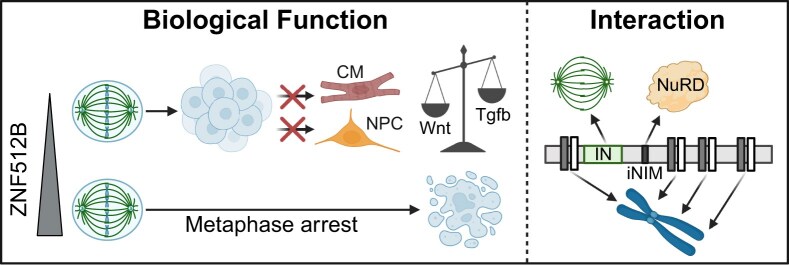

## Introduction

Zinc finger (ZF) proteins represent one of the largest protein families in eukaryotes and are characterized by conserved sequence motifs that fold in small ordered domains stabilized by coordinated zinc ions [1, 2]. These domains often mediate nucleic acid binding [3–5, 6], enabling many ZF proteins to function as sequence-specific transcription factors that regulate gene expression during development, differentiation, and cell cycle progression [1]. Beyond their classical role in transcriptional regulation, a growing number of ZF proteins have been found to exert non-transcriptional functions, including direct involvement in RNA metabolism, chromatin remodeling, protein homeostasis, and even cytoskeletal regulation [1, 3, 7]. Recent studies have demonstrated that certain ZF proteins can also localize to mitotic structures such as the spindle apparatus, where they may contribute to chromosome segregation and mitotic fidelity independently of any DNA-binding activity [8, 9]. These findings suggest that ZF proteins are functionally versatile regulators involved in both nuclear and cytoplasmic processes essential for maintaining genome stability and cellular homeostasis.

Previously, using label-free quantitative mass spectrometry (lf-qMS) screens we identified ZNF512B to be associated with the evolutionary conserved histone variant H2A.Z [10, 11], as well as with H2A.Z’s binding partners PWWP2A [11] and HMG20A [12]. ZNF512B, formerly known as GAM/ZFp [13], is a vertebrate-specific ZF protein. It contains eight ZF domains, which are arranged in four pairs that each comprise one atypical C2HC followed by one typical C2H2 sequence. ZNF512B also contains a large internal region (I) with low complexity and a nuclear localization signal (NLS). Only a few studies of ZNF512B function are available so far; these demonstrate a role for the protein in gene regulation and, possibly as a result, in cell homeostasis [13]. Further, ZNF512B and its paralogue ZNF512 were found to initiate heterochromatin formation at repetitive elements and pericentric regions [14]. It has also been suggested that mutations in regulatory sequences of the *ZNF512B* gene may have a role in the neurodegenerative disease amyotrophic lateral sclerosis (ALS) [15, 16, 17].

We recently discovered a conserved amino acid motif within ZNF512B that resembles a consensus sequence known to act as an interaction motif for the nucleosome remodeling and deacetylase (NuRD) complex. Such NuRD-interaction motifs (NIMs) have been previously identified in the N-terminus of the transcription factor Friend of GATA-1 (FOG-1) and several other regulators of gene expression [18, 19, 20]. We demonstrated that this consensus motif, which is more variable in its composition than previously thought, is both sufficient and required for direct and high-affinity RB Binding Protein 4 (RBBP4) interaction and thereby NuRD complex binding [21]. We further identified ZNF512B as a repressor of gene expression that can act in both NuRD-dependent and -independent ways. Surprisingly, high levels of ZNF512B expression lead to chromatin aggregation foci that form independent of ZNF512B’s interaction with the NuRD complex but depend on its ZF domains.

Here, we report another unexpected function for ZNF512B—one that is both zinc finger- and NuRD-independent. We identify ZNF512B as a mitotic spindle-associated factor that regulates metaphase exit. Its internal domain interacts with proteins involved in the process of chromosome segregation and incorporates a mammalian-specific proline-rich region containing 25 repeats of a six-residue motif, that is predicted to form a highly basic, three-faced β-helix and is sufficient for ZNF512B to bind the mitotic spindle. Elevated levels of ZNF512B cause a severe and fatal metaphase arrest, a striking phenotype that primarily depends on ZNF512B’s spindle-binding ability combined with its chromatin tethering function. Conversely, depletion of ZNF512B leads to accelerated stem cell proliferation and impairs the ability of these cells to differentiate into either beating cardiomyocytes (CMs) or neuronal progenitor cells (NPCs), as further evidenced by the deregulation of numerous gene expression programs involved in, among other processes, cell cycle control, neurogenesis, and muscle contraction.

In conclusion, our findings reveal that ZNF512B functions not only as a chromatin aggregator and transcriptional repressor but also as a novel regulator of mitosis through its association with spindle fibers and its interactors. Moreover, ZNF512B plays a critical role in orchestrating stem cell proliferation, migration, and differentiation, highlighting its multifunctional role in coordinating cell division and developmental processes.

## Materials and methods

### Cell culture and transfections

HeLa Kyoto (HeLaK) cells were grown in Dulbecco’s modified Eagle’s medium (DMEM, Gibco) supplemented with 10% (v/v) fetal calf serum (FCS, Gibco) and 1% (w/v) penicillin/streptomycin (37°C, 5% CO_2_) and routinely tested for mycoplasma contamination via polymerase chain reaction (PCR). Transfections of HeLaK cells were performed using FuGENE^®^ HD Transfection Reagent (Promega) according to the manufacturer’s instructions. Unless stated otherwise, cells were harvested by trypsinization for various assays 48 h after transfection. Transfections of mouse embryonic stem cells (mESCs, v6.5 strain) were performed using jetPEI DNA Transfection Reagent (Polyplus) according to the manufacturer’s protocol. Twenty-four hours after transfection, puromycin (0.25 µg/ml) was used to select mESCs for at least 10 days. Finally, mCherry-positive mESC colonies were characterized by genomic PCR, RT-qPCR, and immunoblotting, respectively.

Naïve mESCs were cultivated in 2iLIF condition as previously described [12]. Specifically, naïve mESCs were cultured on six-well plates coated with 0.1% (w/v) gelatine in 2iLIF medium [N2B27 medium supplemented with 50 μM β-mercaptoethanol, 2 mM L-glutamine, 0.1% (w/v) sodium bicarbonate, 0.11% (w/v) bovine serum albumin fraction V, 1000 units/ml recombinant mouse leukemia inhibitory factor (LIF), 1 µM PD0325901 (MEK inhibitor), and 3 µM CHIR99021 (GSK3 inhibitor)]. Every two days, cells were passaged using Accutase (Stemcell) at a density of 1 × 10^5^ cells/well.

Embryoid body (EB)-mediated differentiation into CMs was performed as previously described [12]. Briefly, naïve mESCs (Day 0) were primed using differentiation medium [DMEM, Gibco supplemented with 10% (v/v) FCS, 2 mM L-glutamine, 1% (w/v) nonessential amino acids, 0.1 mM β-mercaptoethanol, and 1000 U/mL LIF] for 2 days. Hanging drops containing 1000 cells in 25 µl of differentiation medium (without LIF, supplemented with 50 µg/ml vitamin C) were prepared and cultured for 4 days. Afterward, droplets containing EBs were carefully transferred to a 24-well plate coated with 0.1% (w/v) gelatine and further cultured for 1.5 days in differentiation medium (supplemented with 50 µg/ml vitamin C). Beating CMs were observed starting from differentiation Day 7.

Neural progenitor cell (NPC) differentiation was carried out based on the protocol by Bibel *et al*. [22]. Briefly, naïve mESCs were primed as mentioned above. Hanging drops containing 1000 cells were prepared in 25 µl of differentiation medium (without LIF) for 4 days. Then, EBs were pooled and cultured on 100/20 mm bacterial plates in suspension in differentiation medium [without LIF, supplemented with 5 µM retinoic acid (RA)] for 4 days. Medium was changed every two days. On differentiation Day 10, the generated EBs containing NPCs were collected for further analyses.

### Plasmid cloning

Human ZNF512B full-length (FL) and deletion plasmids were obtained from [21]. Additional GFP-ZNF512B_IN, _IC, _ΔIN, and _IN_ΔNLS plasmids were constructed as described in [21]. The SV40 large T-antigen NLS sequence was introduced to the IN construct to ensure proper nuclear localization. *Xenopus laevis* Znf512b was amplified from *X. laevis* complementary DNA (cDNA) using Q5 High-Fidelity DNA Polymerase (NEB) and cloned into the pIRESneo-GFP [23] vector via NEBuilder HiFi DNA Assembly (NEB). pc1116-H2B-mRFP [24] was a gift from Prof. M. Cristina Cardoso (Cell Biology and Epigenetics, Department of Biology, Technical University of Darmstadt, Germany). *Zfp512b* KO mESC cell lines were established using CRISPR–Cas9 technology. Briefly, single guide RNAs (sgRNAs) were designed to target the third exon of *Zfp512b* using the online tool CRISPOR, synthesized by Integrated DNA Technology (IDT), and cloned into the vector pX461 (Addgene). Donor pUC19-based vectors containing either mCherry or a puromycin resistance and mammalian transcriptional triple terminators bGH + hGH + SV40 (synthesized by GENEWIZ) surrounded by homology arms were constructed via NEBuilder HiFi DNA Assembly (NEB). Homology arms were generated from mESC genomic DNA obtained using the QIAamp DNA Mini Kit (QIAGEN) via PCR using Q5 High-Fidelity DNA Polymerase (NEB). All relevant sgRNA sequences and primers are listed in the Supplementary Information.

### Primers and oligos

All primers and oligos are listed in the Supplementary Information.

### Immunofluorescence microscopy

Immunofluorescence (IF) staining of cells and their microscopic analysis was performed as previously described [12]. Briefly, 1 × 10^5^ adherent HeLaK cells expressing GFP, GFP-ZNF512B, or its deletions/mutants were seeded on coverslips in 24-well plates and cultured overnight (ON). The next day, cells were washed two times with phosphate-buffered saline (PBS) and fixed for 15 min in PBS containing 1% (v/v) formaldehyde. After washing, cells were permeabilized and blocked with PBS containing 0.1% (v/v) Triton^™^ X-100 (PBS-T) and 1% (w/v) bovine serum albumin (BSA) for 20 min. Cells were incubated stepwise with primary and then secondary antibodies in PBS-T + 1% (w/v) BSA for 30 min with three wash-steps using PBS-T in between. After three final PBS-T wash-steps, DNA was stained with 10 µg/ml Hoechst solution for 3 min. After washing with H_2_O, coverslips with cells were then mounted in Fluoromount-G^®^ mounting medium (SouthernBiotech) on microscope slides (Epredia). Images were acquired using an Axio Observer.Z1 inverted microscope (Carl Zeiss) with an Axiocam 506 mono camera system. Image processing was performed with Zeiss Zen (version 3.1, blue.edition) and ImageJ2 (version 2.14.0/1.54f).

Live-cell imaging to monitor mitotic progression of HeLaK cells expressing RFP-H2B and various GFP-ZNF512B constructs was executed with a CELLCYTE X (Cytena) microscope using a ×10 objective. Cells were transfected as described above. Imaging started 4–6 h post-transfection with the following set up: 16 images per well were taken every 20 min for 36 h with 150 ms exposure time for the green channel (excitation: 473–492 nm, emission: 502–561) and 450 ms exposure time for the red channel (excitation: 580–598, emission: 612–680 nm). Image processing was performed with Zeiss Zen (version 3.1, blue.edition), ImageJ2 (version 2.14.0/1.54f), and Imaris (version 9.9.0). For more detailed 3D visualization, a Zeiss Spinning-Disk microscope system (Axio Observer.Z1) equipped with a ×100 objective was used.

For growth curve analyses of mESCs, cells were imaged every 1 h for 3 days and 25 pictures/well were taken at each time point. Live-cell imaging movies and analyses of growth curves were generated with the Cytena software.

### Antibodies

All antibodies are listed in the Supplementary Information.

### DNase I-immunoprecipitation (DNase I-IP) and mass spectrometry sample preparation

Immunoprecipitation was performed in quadruplicates using GFP-Trap^®^ magnetic particles M-270 (Chromotek) following the manufacturer’s protocol. In brief, 5 × 10^6^ cells per replicate were incubated for 30 min on ice with 200 µl of RIPA buffer [10 mM Tris/Cl pH 7.5, 150 mM NaCl, 0.5 mM EDTA, 0.1% (v/v) SDS, 1% (v/v) Triton^™^ X-100, 1% (w/v) deoxycholate] supplemented with DNase I (187.5 U/ml, Thermo Fisher Scientific), 2.5 mM MgCl_2_, and protease inhibitor cocktail. After centrifugation at 17 000 × *g* for 10 min at 4°C, lysate was transferred to a fresh tube and mixed with 300 µl of dilution buffer (10 mM Tris/Cl pH 7.5, 150 mM NaCl, and 0.5 mM EDTA) supplemented with protease inhibitor cocktail. Lysate was then incubated with 25 µl of GFP-Trap^®^ magnetic particles M-270 (Chromotek) rotating overnight at 4°C. After incubation, beads were washed three times with wash buffer [10 mM Tris/Cl pH 7.5, 150 mM NaCl, 0.05% (v/v) Nonidet^™^  *P*-40 Substitute, and 0.5 mM EDTA].

For analysis via mass spectrometry, precipitated proteins were eluted by incubation of beads with 50 µl of elution buffer [2 M Urea, 50 mM Tris/Cl pH 7.5, 2 mM DTT, and 20 µg/ml Trypsin Gold (Promega)] for 30 min at 37°C and 1400 rpm in the dark. Supernatants were transferred into fresh tubes, and beads were further incubated in 50 µl of alkylation buffer (2 M Urea, 50 mM Tris/Cl pH 7.5, and 10 mM chloroacetamide) for 5 min at 37°C and 1400 rpm in the dark. Both eluates were combined and incubated overnight at 25°C and 800 rpm in the dark. The next day, trypsin digestion was stopped by addition of 1 µL trifluoroacetic acid (Thermo Fisher Scientific).

### Label-free quantitative mass spectrometry and protein–protein interaction network analysis with STRING

lf-qMS data acquisition and analysis were performed as previously described with some modifications [21]. Briefly, peptides were desalted on StageTips [24] and analyzed by nanoflow liquid chromatography using an NanoElute2 HPLC system coupled online to a TimsTOF HT mass spectrometer (both Bruker). Peptides were separated on an Aurora Ultimate CSI 25 × 75 C18 UHPLC column (Ionopticks) at 50°C for reverse-phase chromatography using a 37 min gradient from 2% to 32% (v/v) acetonitrile in 0.1% (v/v) formic acid at 300 nl/min. MS data were acquired with the standard data-dependent acquisition PASEF mode.

Raw files were processed with MaxQuant (v2.4.2.0) [25] against the human UniProt database (81 194 entries). Carbamidomethylation was set as a fixed modification, while methionine oxidation and protein *N*-acetylation were included as variable modifications. Results were determined at a false discovery rate of 1% on peptide and protein level and LFQ quantification enabled. Reverse hits, contaminants, site-only identifications, and proteins identified with fewer than two peptides (including at least one unique peptide) were excluded from further analysis. Missing values were imputed using a beta distribution at the limit of quantification.

Protein–protein interaction (PPI) networks were obtained from STRING (version 12.0) [26] database with “confidence score” > 0.7 (high confidence). Clusters were generated with *k*-means (obsolete) choosing the option of three clusters. The PPI enrichment *P*-value was < 1.0e-16.

### RNA extraction, RT-qPCR, and RNA-seq

RNA extraction was performed as described [27]. Briefly, total RNA was extracted using the RNeasy Mini Kit (QIAGEN) with on column DNase digest (RNase-Free DNase Set, QIAGEN) according to the manufacturer’s instructions. One microgram of total RNA was reverse transcribed using the Transcriptor First Strand cDNA Synthesis Kit (Roche) with random hexamer primers, according to the manufacturer’s protocol. For subsequent qPCR analysis, technical triplicates of 3 µl of cDNA (1:20 dilution), 7.5 µl of iTaq^™^ Universal SYBR^®^ Green Supermix (Bio-Rad), 3 µl of water, and 1.5 µl of primer mix (5 µM forward and reverse primer) were used. The qPCR program consisted of 5 min at 95°C, followed by 40 cycles at 95°C for 3 s and 60°C for 20 s. Lastly, Ct values of technical triplicates were averaged, and fold change expression was calculated with the Delta-Delta Ct method, normalizing to control samples and HPRT1 expression.

mRNA sequencing (mRNA-seq) was performed at Biomarker Technologies (Germany).

### Bioinformatics analysis

mRNA-seq analysis was performed on the Galaxy platform [28]. Raw sequencing files (FASTQ) were adapter- and quality-trimmed using trimGalore [29]. Alignment of the trimmed sequencing reads to the mm9 reference genome (Illumina’s iGenomes) was performed using Hisat2 v.2.2 [30]. Read counts per gene were obtained from BAM files using featureCounts [31] from the Rsubread package [32] and the mouse mm9 gene transfer format (GTF).

Normalization of read counts and detection of differentially expressed genes (DEGs) was performed with DESeq2 [33] v1.46.0 in R v4.4.1 (https://www.R-project.org/). If not otherwise indicated, significant DEGs were chosen based on an adjusted *P*-value < 0.05 (Supplementary Tables S1 and S2). Based on the resulting DEGs, Venn diagrams were generated using the eulerr [34] and ggplot2 [35] packages. The overlap between KO clones and cell stages was used for an over-representation gene ontology (GO) [36, 37] and KEGG pathway [38, 39, 40] analysis with clusterProfiler [41]. Volcano plots were created with ggplot2 [35]. For the analysis of lf-qMS data, volcano plots were generated using ggplot2 based on thresholds of −log10(*P*-value) > 1.3 (two-tailed Welch’s *t*-test) and log2 fold change > 1. Genes encoding significantly enriched proteins were subjected to a GO ORA [36, 37] using clusterProfiler [41].

### Structure predictions

Protein structures were predicted using either AlphaFold2 implemented as ColabFold (https://colab.research.google.com/github/sokrypton/ColabFold/blob/main/AlphaFold2.ipynb) or the AlphaFold3 server (https://alphafoldserver.com/) and visualized in Pymol 3.1 (Schroedinger software).

## Results

### ZNF512B localizes to mitotic spindles via its internal region and independent of NuRD interaction

**Figure 1.**
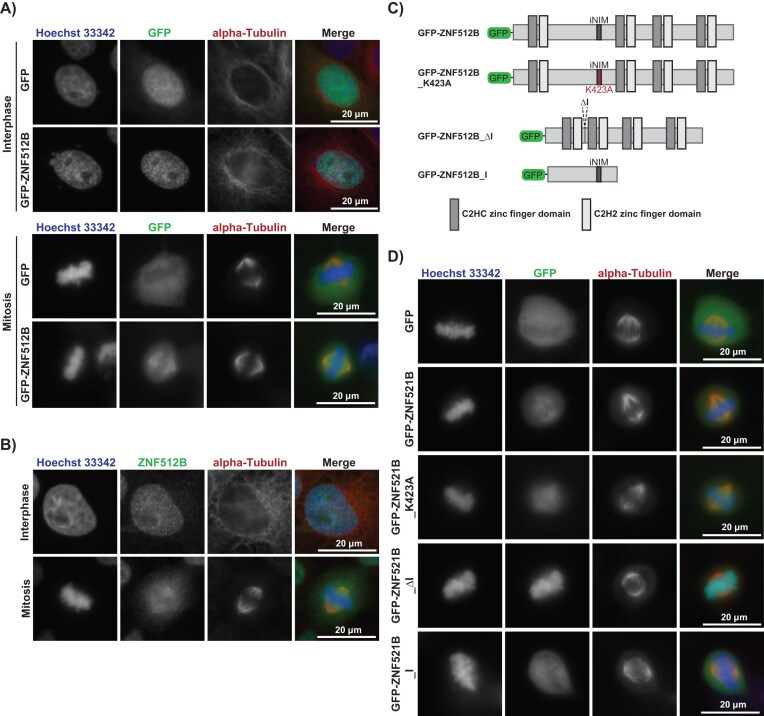
ZNF512B associates with mitotic spindles via its internal region, independently of NuRD binding. (**A**) IF microscopy pictures of HeLaK cells during interphase (top) and mitosis (metaphase; bottom) transfected with GFP (control) or GFP-ZNF512B (green). DNA (blue) was visualized by Hoechst 3342 and microtubules/mitotic spindles by anti-alpha-tubulin (red) staining; scale bar: 20 µm. See also Movie 1. (**B**) IF microscopy pictures of HeLaK cells stained with Hoechst 3342 (DNA, blue), anti-ZNF512B (green), and anti-α-tubulin (microtubules/mitotic spindle; red); scale bar: 20 µm. (**C**) Schematic depiction of GFP-ZNF512B, its lysine 423 to alanine substitution mutant (K423A) and various deletion constructs. (**D**) IF microscopy pictures of HeLaK cells during metaphase transfected with GFP (control), GFP-ZNF512B or point mutant or deletion constructs (green). DNA (blue) was visualized by Hoechst 3342 and microtubules/mitotic spindles by anti-alpha-tubulin (red) staining; scale bar: 20 µm.

We recently reported that overexpression of the zinc finger protein ZNF512B, fused either N- or C-terminally to a GFP-tag (GFP-ZNF512B or ZNF512B-GFP), leads to the formation of distinct nuclear foci, the size of which correlates with ZNF512B expression levels [21]. This chromatin aggregation phenotype is driven by a combination of ZNF512B’s zinc finger-dependent abilities to oligomerize and to bind DNA. Upon closer examination of GFP-ZNF512B expressing cells outside of interphase, we observed a striking shift in its subcellular localization: In metaphase, a large portion of the GFP-ZNF512B protein pool re-localized from chromatin to the mitotic spindle, with only a minor fraction remaining associated with condensed chromosomes (Fig. 1A and Supplementary Fig. S1A). This spindle localization was further confirmed in greater detail by live-cell imaging of GFP-ZNF512B in cells transiently expressing red fluorescent protein (RFP)-tagged histone H2B to visualize chromatin (Movie 1).

Given the strong overexpression of GFP-ZNF512B protein in our experimental system (approximately 500-fold enrichment of ZNF512B mRNA compared to endogenous levels [21]), we next asked whether the observed mitotic spindle localization might represent an overexpression artifact. To address this question, we performed IF microscopy with an antibody against endogenous ZNF512B [21]. Consistent with the localization of overexpressed GFP-ZNF512B, endogenous ZNF512B protein was also clearly detected at the mitotic spindle apparatus in metaphase, anaphase and at the midbody in cytokinesis (Fig. 1B, and Supplementary Fig. S1B and C). This finding confirms that ZNF512B’s association with spindle fibers during mitosis is not an experimental artifact.

Next, we investigated which region in ZNF512B is required for spindle binding by analyzing the mitotic localization of several GFP-ZNF512B mutant constructs that we had previously functionally described [21] (Fig. 1C). A key focus was to assess whether spindle recruitment requires ZNF512B’s direct interaction with the NuRD complex, which is mediated by its variant internal NuRD interaction motif (iNIM) [21]. Several components of NuRD, such as CHD4 [42] and MTA1 [43], have been reported to localize to the mitotic spindle, and the NuRD subunit RBBP4 (to which ZNBF512B directly binds) has been shown to be essential for bipolar spindle assembly during meiotic metaphase [44].

To evaluate the potential role of the NuRD complex in recruiting ZNF512B to the mitotic spindle, we used the K423A point mutant of ZNF512B (Fig. 1C), which is unable to bind NuRD [21]. We also examined GFP-ZNF512B constructs with either all eight zinc fingers deleted—retaining only the internal region (I)—or with the internal region containing the iNIM deleted—retaining all zinc fingers (ΔI) (Fig. 1C). Unexpectedly, both GFP-ZNF512B_K423A and GFP-ZNF512B_I mutants retained spindle-binding ability (Fig. 1D). In contrast, GFP-ZNF512B_ΔI failed to associate with the spindle apparatus (Fig. 1D), indicating that the internal region of ZNF512B is both necessary and sufficient for mitotic spindle localization, and that this interaction is NuRD independent.

Next, we investigated whether ZNF512B is responsible for recruiting NuRD complex members to the mitotic spindle. Overexpression of GFP-ZNF512B_I and GFP-ZNF512B_ΔI constructs revealed that the relatively weak localization of endogenous CHD4 to spindle fibers remained, regardless of the ZNF512B’s ability to bind the NuRD complex (Supplementary Fig. S1D). Interestingly, ZNF512B’s direct binding partner RBBP4 showed increased localization to spindle fibers when ZNF512B’s internal region containing the iNIM was present, compared to the ΔI construct, which is unable to associate with either the mitotic spindle or RBBP4 (Supplementary Fig. S1E). These results suggest that ZNF512B may not be required for the recruitment of all NuRD complex components to the spindle apparatus, but does play a role in the spindle association of its direct interaction partner RBBP4.

In summary, we have identified the internal region of ZNF512B to be necessary and sufficient for its association with mitotic spindles, independently of NuRD complex interaction.

### ZNF512B’s N-terminal internal region is sufficient for spindle interaction

**Figure 2.**
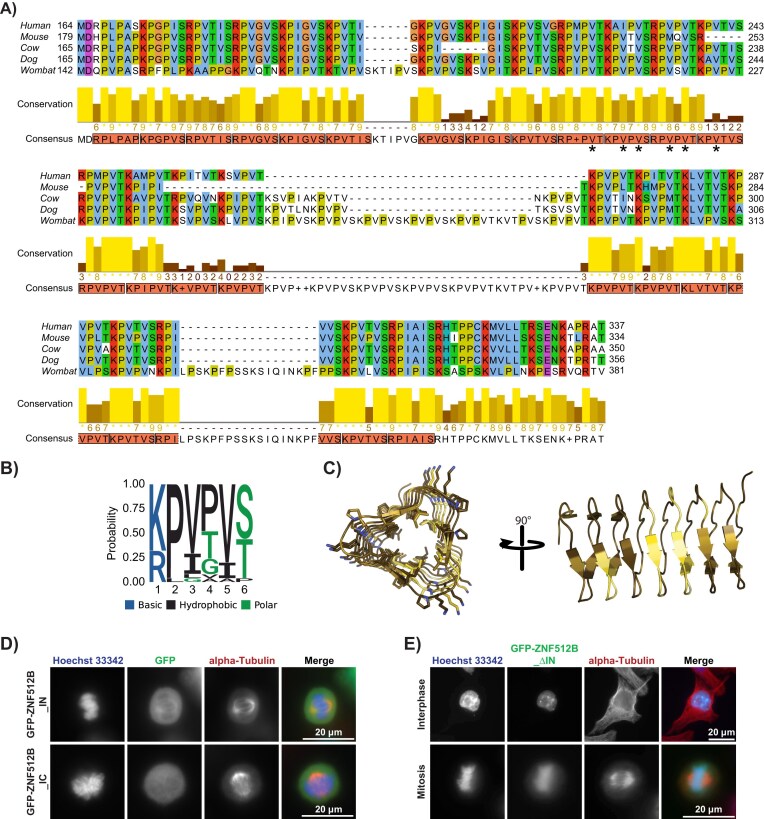
The mammalian-specific N-terminal portion of ZNF512B’s internal region forms a β-helix structure and is sufficient and required for spindle association. (**A**) Amino acid alignment of the N-terminal portion of ZNF512B’s internal region (IN) across mammalian and marsupial (Wombat) species with the SnapGene (Version 8.0.3) Software using Clustal-Omega and Jalview (Version 2.11.4.1). Protein Sequences were obtained from uniprot.org for human (*Homo sapiens*, Entry Q96KM6), mouse (*Mus musculus*, Entry Q6ZPW1), cow (*Bos taurus*, Entry G3N139), dog (*Canis lupus familiaris*, Entry A0A8P0NRY1), and wombat (*Vombatus ursinus*, Entry A0A4 × 2L7 × 1). Consensus sequence and conservation score were generated using Jalview Version 2.11.4.1. Amino acids are colored in the Clustal X default coloring scheme: hydrophobic (blue), positive charge (red), negative charge (magenta), polar (green), cysteine (pink), glycine (orange), proline (yellow), aromatic (cyan), and unconserved or gap (white). Repeat regions are marked by oranges boxes in the consensus sequence. (**B**) Sequence logo of tandem repeat motifs within the IN region of ZNF512B. Residue probability at each position is represented by height. The logo was generated using the ggseqlogo (Version 0.2.2) package in R [45]. (**C**) AlphaFold2 prediction of the folding of ZNF512B’s IN region. Shown are two views rotated 90°. (**D**) IF microscopy pictures of HeLaK cells during metaphase transfected with GFP-ZNF512B_IN or GFP-ZNF512B_IC constructs (green). DNA (blue) was visualized by Hoechst 3342 and microtubules/mitotic spindles by anti-α-tubulin (red) staining; scale bar: 20 µm. (**E**) IF microscopy pictures of HeLaK cells during interphase (top) or metaphase (bottom) transfected with GFP-ZNF512B_ΔIN constructs (green). DNA (blue) was visualized by Hoechst 3342 and microtubules/mitotic spindles by anti-α-tubulin (red) staining; scale bar: 20 µm.

To further refine the region of ZNF512B responsible for spindle binding, we first examined the evolutionary conservation of ZNF512B’s internal region, which comprises a low-complexity sequence [21]. Interestingly, only a C-terminal portion of the internal region (IC) is conserved across all vertebrates, whereas an N-terminal portion (IN) is specific to mammals (Supplementary Fig. S2). Visual inspection of the sequence of ZNF512B’s IN region revealed 25 tandem copies of a six-residue motif that broadly follows the consensus sequence [KR]-P-ϕ-[Pπ]-ϕ-π (where ϕ is an aliphatic hydrophobic residue and π is a small, polar residue; Fig. 2A and B). This region was conserved overall in other mammalian orthologues, though with the insertion or deletion of one or more repeat motifs compared to the human protein. Prediction of the structure of the human IN region using AlphaFold2 suggests, albeit with moderate confidence, that this region forms an unusual three-faced, right-handed β-helix (Fig. 2C and Supplementary Fig. S3A).

Based on these sequence alignments, we generated GFP-tagged constructs containing either the IN or IC region of ZNF512B; with the IN region fused to a NLS sequence to ensure nuclear localization of both constructs as the endogenous NLS is predicted to be located solely within the IC domain (Supplementary Fig. S2). Surprisingly, upon transient transfection, only the mammalian-specific IN but not the evolutionary conserved IC region was able to associate with spindle fibers during metaphase (Fig. 2D). Equally, deletion of the IN region within full-length ZNF512B (GFP-ZNF512B_ΔIN) resulted in the inability to bind mitotic spindles (Fig. 2E). These data clearly demonstrate that the IN region in ZNF512B is sufficient and required for spindle interaction.

In line with this finding, GFP-tagged *X. laevis* Znf512b protein, which naturally lacks the IN domain (Supplementary Fig. S2), did not localize to the mitotic spindle nor was it found in the midbody, although it was still able to bind condensed chromatin during mitosis and to form ZF-dependent chromatin aggregates in interphase, when expressed in human HeLaK cells (Supplementary Fig. S3B).

In conclusion, we identified a mammalian-specific internal N-terminal region of ZNF512B, which may adopt a β-helix structure, as both necessary and sufficient for its interaction with the mitotic spindle.

### ZNF512B’s internal region interacts with proteins involved in chromosome segregation

**Figure 3.**
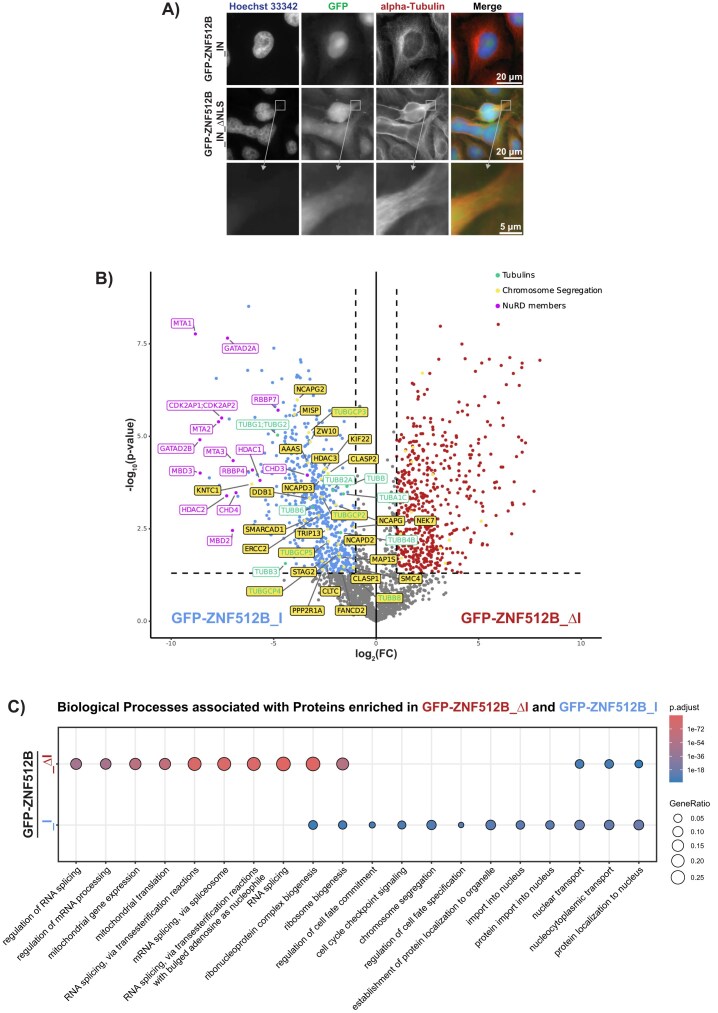
ZNF512B’s internal domain mediates interaction with proteins involved in chromosome segregation. (**A**) IF microscopy pictures of HeLaK cells during interphase transfected with GFP-ZNF512B_IN or GFP-ZNF512B_IN_ΔNLS constructs (green). DNA (blue) was visualized by Hoechst 3342 and microtubules by anti-α-tubulin (red) staining; scale bar: 20 µm. Bottom panel shows an enlargement (gray box) of cytoplasmic microtubules and GFP-ZNF512B_IN_ΔNLS, demonstrating no association between these two structures; scale bar: 5 µm. (**B**) Volcano plot of lf-qMS data comparing proteins enriched on GFP-ZNF512B_I (blue) with those bound to GFP-ZNF512B_ΔI (red). Yellow dots mark proteins associated with the process of chromosome segregation, green dots mark tubulin proteins and purple dots mark NuRD complex members. Dashed lines indicate thresholds for significantly enriched proteins (*P*-value < 0.05, log2FC > 1). (**C**) ORA using the GO database (“biological processes”) of significantly enriched proteins identified by lf-qMS (see B) bound to either GFP-ZNF512B_I or GFP-ZNF512B_ΔI.

Next, we investigated how ZNF512B associates with mitotic spindles. One possibility is a direct interaction with tubulin. We expressed the IN domain without an extra NLS to prevent its nuclear localization (GFP-ZNF512B_IN_ΔNLS), thereby enabling a potential interaction with microtubules in interphase cells if this domain were capable of binding non-mitotic tubulin fibers. IF microscopy pictures clearly confirmed cytoplasmic localization of this ΔNLS GFP-tagged IN construct; however, no co-localization with microtubules was observed (Fig. 3A). These findings indicate that ZNF512B either does not directly bind tubulin or that mitosis-specific post-translational modifications of tubulin (or ZNF512B) or additional proteins are required to mediate this interaction.

To identify potential candidate proteins that mediate ZNF512B’s spindle association, we reanalyzed our previously published label-free quantitative mass spectrometry (lf-qMS) dataset of proteins interacting with GFP-ZNF512B and the NuRD-binding deficient GFP-ZNF512B_K423A mutant [21]. Interestingly, STRING analysis of all proteins identified in these pull-downs (*P*-value < 0.05, log2FC > 1) revealed that several proteins, interacting with ZNF512B independently of NuRD, are also linked, besides ribosome biogenesis, to chromosome segregation (Supplementary Fig. S4A and B).

To further refine the candidate list, we performed an additional lf-qMS experiment comparing proteins interacting with the ZNF512B internal domain alone (GFP-ZNF512B_I; sufficient for spindle binding) to proteins associated with a ZNF512B construct lacking the internal domain (GFP-ZNF512B_ΔI, unable to bind to the mitotic spindle). In this experiment, samples for GFP-Trap immunoprecipitations were prepared using DNase I-digested whole-cell lysates instead of MNase-digested nuclear extracts employed previously [21], enabling the pull-down of a substantially larger number of proteins (Supplementary Fig. S4C and D). As expected, comparison of proteins enriched in GFP-ZNF512B_I versus GFP-ZNF512B_ΔI (Fig. 3B) again revealed, by over-representation analysis (ORA) using the GO database of biological processes, enrichment of proteins associated with chromosome segregation specifically for the GFP-ZNF512B_I construct, but not for GFP-ZNF512B_ΔI samples (Fig. 3C). Interestingly, several β-tubulin and γ-tubulin isoforms, likely constituents of the mitotic spindle, were also identified. Together, these results suggest that the internal domain of ZNF512B is sufficient for interaction with the mitotic spindle and spindle-associated proteins.

### Overexpression of GFP-ZNF512B results in mitotic arrest dependent on IN-mediated spindle binding

**Figure 4.**
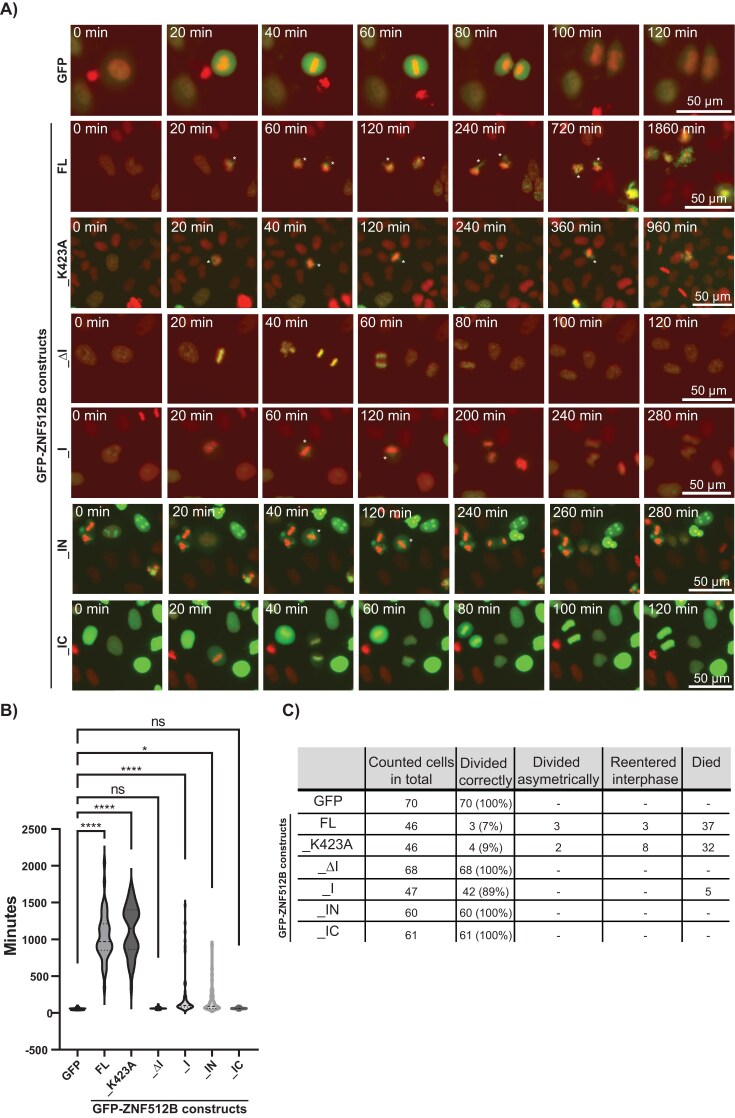
Elevated levels of ZNF512B lead to severe mitotic delay, depending on its IN region. (**A**) Pictures taken at different time points from live-cell imaging movies from HeLaK cells stably expressing RFP-H2B (red) transfected with various GFP-ZNF512B mutant and deletion constructs (green, see Fig. 1C). Asterisks indicate mitotic spindles. See Movies 2–8; scale bar: 50 µm. (**B**) Quantification of time spent in mitosis by transfected HeLaK cells (see A). Significance was calculated in Prism with one-way ANOVA, where ns = >0.05, * = <0.05, ** = <0.01, *** = <0.001, and **** = <0.0001. For total cell number see panel (C). (**C**) Table summarizing observed phenotypes in transfected cells (see A).

Next, we investigated whether elevated levels of ZNF512B and/or its spindle-binding ability affect mitotic progression. We transfected either GFP alone (control) or diverse GFP-ZNF512B mutant constructs into HeLaK cells stably expressing RFP-tagged H2B and performed live-cell imaging. Cells were monitored over a 36 h time period, with images acquired every 20 min using the CELLCYTE X (Cytena) system. The duration of mitosis was subsequently quantified. Typically, HeLaK cells spend approximately one hour in mitosis, as we also observed in cells expressing GFP alone (Fig. 4A–C, Movie 2). In contrast, GFP-ZNF512B overexpression caused a severe mitotic arrest lasting up to 25 h, with an average mitotic duration of 17 h, after which most of the cells died (Fig. 4A–C, Movie 3). We next tested cells expressing the GFP-ZNF512B_K423A point mutant, which cannot bind NuRD but retains the ability to associate with the mitotic spindle. These cells exhibited a similarly pronounced mitotic arrest followed by cell death, indicating that this phenotype is independent of NuRD binding (Fig. 4A–C, Movie 4).

On the other hand, deletion of the spindle-binding internal domain (GFP-ZNF512B_ΔI) did not cause any mitotic defects (Fig. 4A–C, Movie 5), while expression of only the internal domain alone (GFP-ZNF512B_I) again led to a mitotic delay, though less severe than observed with the full-length protein (Fig. 4A–C, Movie 6). In this case, cells were able to survive a delayed mitotic exit, suggesting that excess of ZNF512B and its enhanced spindle association and zinc finger-mediated chromatin binding are particularly detrimental. As expected, overexpression of spindle-binding GFP-ZNF512B_IN (Fig. 4A–C, Movie 7), but not GFP-ZNF512B_IC (Fig. 4A–C, Movie 8), also led to a mild mitotic delay of approximately 2.5 h, further supporting the idea that docking of even a minimal ZNF512B fragment with the mitotic spindle apparatus is sufficient to impair timely mitosis exit.

Together, these data suggest that high levels of ZNF512B protein disrupt normal mitotic progression, likely through its combined association with the mitotic spindle and chromatin; interactions that are mediated by the internal N-terminal region and the zinc finger domains, respectively.

### ZNF512B depletion leads to a growth advantage and upregulation of genes involved in cell cycle control

**Figure 5.**
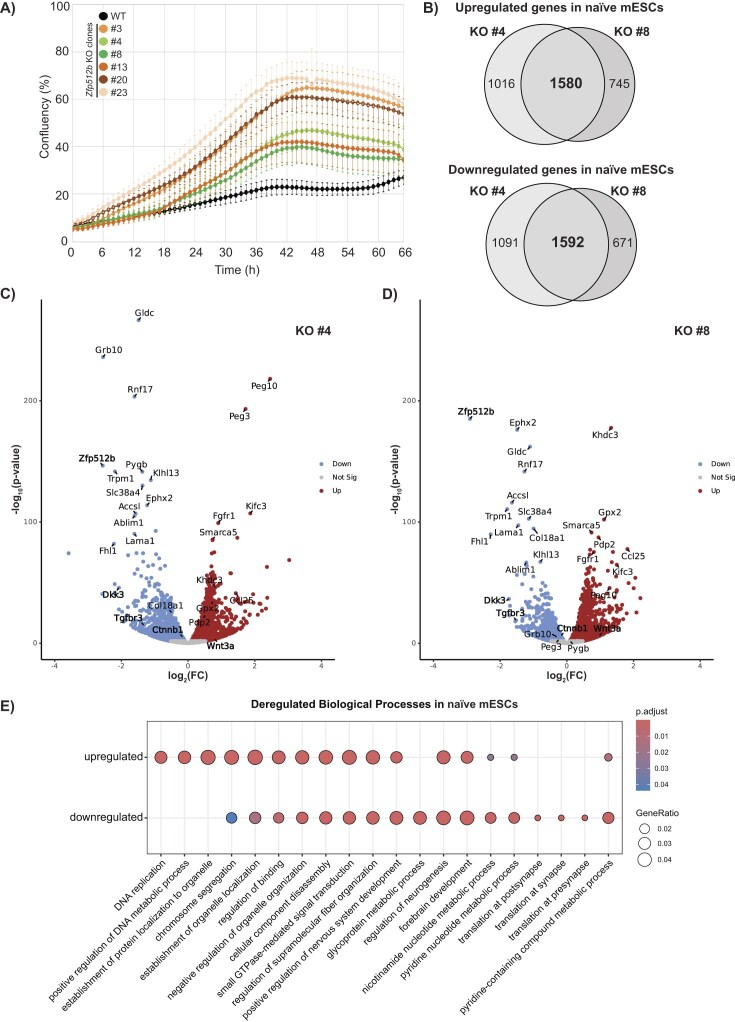
ZNF512B depletion leads to faster proliferating stem cells. (**A**) Growth curve analysis of WT and six naïve *Zfp512b* KO mESC cell clones. Cells were imaged every 1 h for 3 days and 25 pictures/well were taken at each time point. Error bars show standard deviation. (**B**) Venn diagrams of significantly (*P*-value < 0.05) differentially up- or downregulated genes in naïve *Zfp512b* KO mESC clones #4 and #8 compared to naïve WT mESCs. (**C** and **D**) Volcano plots of deregulated genes in naïve *Zfp512b* KO mESC clone #4 or #8 compared to naïve WT mESCs. Significantly (*P*-value < 0.05) downregulated genes in blue, significantly upregulated in red. (**E**) ORA using GO database (“biological processes”) of significantly (*P*-value < 0.05) deregulated genes in both naïve *Zfp512b* KO mESC clones compared to naïve WT mESCs.

Having shown that ZNF512B binds to the mitotic spindle via its internal N-terminal region and that high levels of ZNF512B cause a severe mitotic delay followed by cell death, we next investigated whether depletion of ZNF512B would have the opposite effect on cell division and proliferation. We generated ZNF512B-depleted mouse embryonic stem cells (*Zfp512b* KO mESCs) by inserting a triple transcriptional terminator sequence after the third exon of the murine *Zfp512b* gene (Supplementary Fig. S5A). Upstream of the terminator sequence, an mCherry or puromycin resistance gene were also inserted to enable selection of homozygous clones. It was impractical and technically challenging to establish WT cells subjected to the same genome editing and selection process. We obtained six independent *Zfp512b* KO mESC clones, confirming their identity at the genomic, RNA, and protein levels (Supplementary Fig. S5B–D). Interestingly, all naïve *Zfp512b* KO mESC clones exhibited enhanced proliferation compared to wild-type (WT) cells (Fig. 5A). This striking finding aligns well with our observation that elevated ZNF512B levels cause a mitotic delay, further supporting a role for ZNF512B in the regulation of mitosis in both human and mouse cells.

To gain insight into the molecular changes underlying these proliferative differences, we performed mRNA-sequencing (mRNA-seq) of WT mESCs and two *Zfp512b* KO clones (#4 and #8) in the naïve state (Supplementary Fig. S5E and Supplementary Table S1). These two cell clones, which exhibited the least increased proliferation phenotype, were selected to minimize confounding effects arising from potential off-target genomic changes during selection. mRNA-seq analysis revealed >3000 genes that were commonly deregulated in both naïve *Zfp512b* KO clones compared to WT mESCs, with approximately half being upregulated and the other half being downregulated (Fig. 5B–D).

Using all six previously generated naïve *Zfp512b* KO cell clones, these findings were further validated by RT-qPCR for four consistently deregulated genes (Supplementary Fig. S5F), which are involved in the regulation of cell growth and/or differentiation—*Wnt3a* (Wnt family member 3a; upregulated), *Dkk3* (Dickkopf WNT signaling pathway inhibitor 3; downregulated), *Tgfbr3* (transforming growth factor β receptor type 3; downregulated), and *Ctnnb1* (Catenin β 1; β-catenin signaling; slightly downregulated).

ORA, using the GO database of biological processes, of these commonly deregulated genes revealed that downregulated genes were associated with neurogenesis, while both down- and upregulated genes were linked to metabolism and organelle organization (Fig. 5E). Interestingly, and consistent with our observation that naive *Zfp512b* KO mESCs proliferate faster than WT, upregulated genes were associated with cell cycle regulation via the term “DNA replication” (Fig. 5E).

Together, these data suggest that reduced ZNF512B levels increase cell proliferation and upregulate expression of genes involved in cell cycle progression in naïve mESCs.

### Loss of ZNF512B severely affects cell differentiation and proliferation

**Figure 6.**
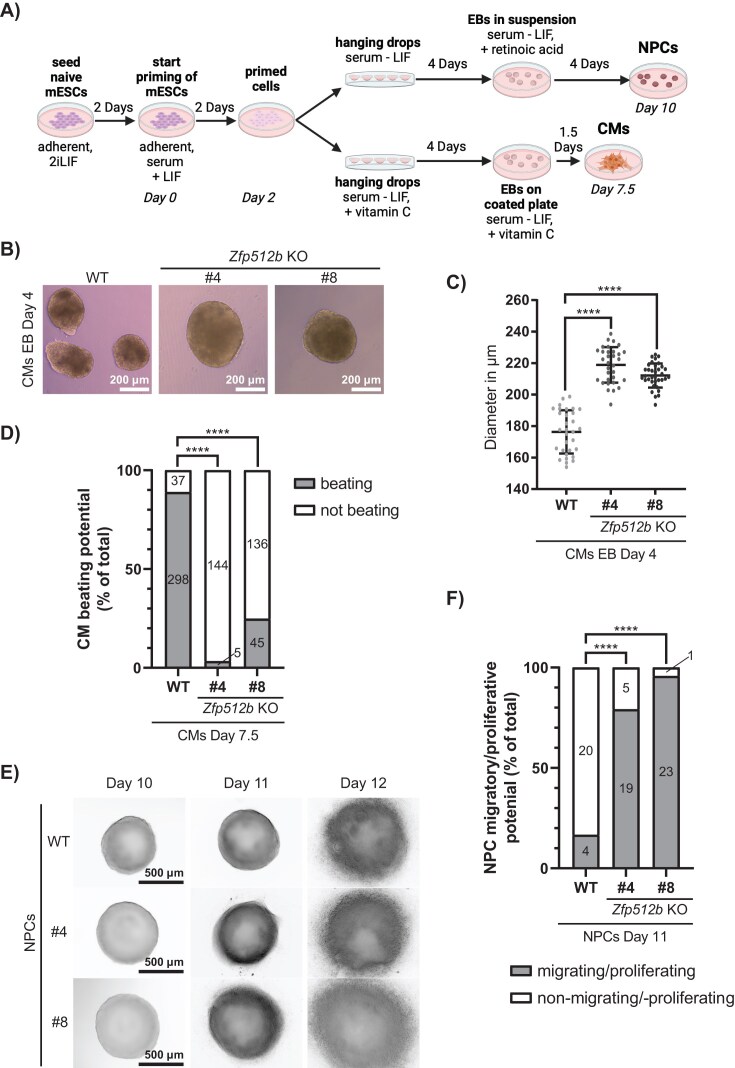
ZNF512B depletion results in differentiation and migration defects. (**A**) Schematic depiction of applied protocols to differentiate mESCs into NPCs (top) or beating CMs (bottom). (**B**) Microscopic images of Day 4 EBs during the differentiation process toward CMs derived from WT or *Zfp512b* KO clone #4 or #8 mESCs; scale bar: 200 µm. (**C**) Quantification of CM-differentiation protocol derived Day 4 EB sizes of WT or *Zfp512b* KO clone #4 or #8 mESCs (see B). 31 WT, 32 #4 and 34 #8 EBs were analyzed and diameters of single EBs depicted in µm. Significance was calculated in Prism with one-way ANOVA, where ns = >0.05, * = <0.05, ** = <0.01, *** = <0.001, and **** = <0.0001. Error bars show standard deviation. (**D**) Beating potential of WT or *Zfp512b* KO clone #4 or #8 EBs at Day 7.5 of the CM differentiation procedure (see A). Numbers in bars indicate counted cells for each condition. Statistical significance was assessed using the chi-square test. Both *Zfp512b* KO clones #4 and #8 differed significantly from WT (*P* < 0.0001). (**E**) Microscopic images of Day 10, 11, and 12 NPCs derived from WT or *Zfp512b* KO clone #4 or #8 mESCs; scale bar: 500 µm. (**F**) Migratory/proliferative potential of NPCs one day after plating Day 10 EBs of WT or *Zfp512b* KO clone #4 or #8 mESCs. Numbers in bars indicate counted EBs for each condition. Statistical significance was assessed using the Fisher’s exact test. Both *Zfp512b* KO clones #4 and #8 differed significantly from WT (*P* < 0.0001).

Given that naïve *Zfp512b* KO mESCs exhibited increased proliferation, we next investigated whether ZNF512B is required for proper mESC differentiation. Using established protocols [12, 22], we differentiated WT mESCs and the two *Zfp512b* KO cell clones #4 and #8 into either beating CMs or NPCs (Fig. 6A). First, we observed that Day 4 EBs derived from both *Zfp512b* KO cell clones were significantly larger than those from WT cells (Fig. 6B and C). Furthermore, *Zfp512b* KO cells failed to properly differentiate into beating CMs (Fig. 6D). Differentiation into NPCs resulted in enlarged cell clusters (Fig. 6E), demonstrating increased proliferation and/or enhanced cell migration (Fig. 6F).

RNA-seq analysis of Day 2 primed cells, as well as differentiated Day 7.5 CMs or Day 10 NPCs (Supplementary Fig. S6A and Supplementary Table S2), identified 203 deregulated genes shared between both *Zfp512b* KO clones compared to WT cells across all three differentiation stages (Supplementary Fig. S6B). The vast majority of these genes were upregulated, with only three genes downregulated (Supplementary Fig. S6C), supporting the notion that ZNF512B primarily functions as a transcriptional repressor, as previously proposed [14, 21]. ORA using the Kyoto Encyclopedia of Genes and Genomes (KEGG) pathway database revealed that commonly upregulated genes were predominantly associated with RNA degradation and, notably, cell cycle regulation (Supplementary Fig. S6D). These findings indicate, once again, that *Zfp512b* KO cells display aberrant cell cycle progression throughout all stages of differentiation.

**Figure 7.**
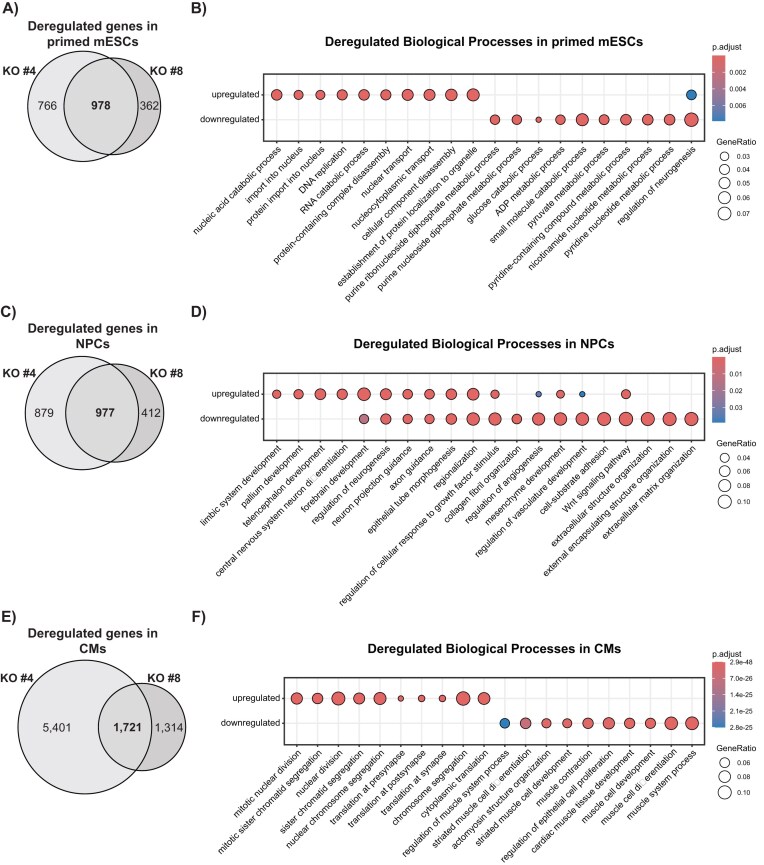
Loss of ZNF512B leads to deregulated gene expression during distinct differentiation pathways. (**A, C**, and **E**) Venn diagrams of significantly (*P*-value < 0.05) DEGs in *Zfp512b* KO mESC clones #4 and #8 compared to WT mESCs in the (A) primed, (C) NPC, or (E) CM stage. (**B, D**, and **F**) ORA using GO database (“biological processes”) of shared significantly (*P*-value < 0.05) deregulated genes in both *Zfp512b* KO clones in the (B) primed, (D) NPC, or (F) CM stage, corresponding to (A), (C), and (E).

Next, we examined gene expression changes at each individual stage of differentiation. In both primed *Zfp512b* KO clones, nearly 1000 genes were differentially expressed compared to primed WT cells (Fig. 7A), with approximately two-thirds being upregulated and one-third being downregulated (Supplementary Fig. S7A).

ORA using the GO database of biological processes revealed that downregulated genes were associated with various metabolic processes, whereas upregulated genes were once again related to processes involved in cell cycle, such as DNA replication, among others (Fig. 7B). A quantitatively similar pattern was observed in Day 10 NPCs (Fig. 7C and Supplementary Fig. S7B), although in this context, the affected genes in *Zfp512b* KO cells were, as expected, primarily linked to neurogenesis (Fig. 7D). Notably, genes involved in the Wnt signaling pathway were also deregulated, corroborating our findings in naïve cells (see Supplementary Fig. S5F) and indicating that this key developmental pathway is disrupted early in development and remains affected during NPC differentiation.

The greatest number of commonly affected genes in both Znf512b KO clones was detected in Day 7.5 CMs (Fig. 7E), with 750 genes being upregulated and 971 being downregulated (Supplementary Fig. S7C). ORA analysis showed that downregulated genes were significantly enriched in terms related to muscle contraction/differentiation and cardiac muscle development (Fig. 7F), consistent with our finding that loss of ZNF512B impairs the differentiation of functional, beating CMs (see Fig. 6D). Interestingly, the upregulated genes were again predominantly associated with cell cycle processes, including mitotic nuclear division and sister chromatid segregation (Fig. 7F).

Collectively, these findings underscore the essential role of ZNF512B in regulating cell proliferation, and in ensuring proper stem cell differentiation.

## Discussion

In this study, we demonstrate that ZNF512B binds to mitotic spindle fibers in a manner that is independent of its interactions with the NuRD complex and with DNA/chromatin. This association with main components(s) of the mitotic spindle apparatus is mediated by a mammalian-specific internal N-terminal region that contains a repeat motif predicted to form a β-helix structure. Elevated ZNF512B protein levels result in a pronounced delay in metaphase exit, ultimately leading to cell death. This severe phenotype depends on ZNF512B’s ability to bind both mitotic spindles and chromatin. In contrast, ZNF512B depletion accelerates stem cell proliferation, impairs stem cell differentiation, and induces the upregulation of genes involved in cell cycle control.

ZNF512B’s ability to bind the mitotic spindle does not depend on its interaction with the NuRD complex, although several NuRD components, such as CHD3 [46], CHD4 [42], MTA1 [43], and RBBP4 [44], have also been reported to localize to the spindle apparatus or to cause spindle disorganization when depleted. Moreover, ZNF512B is likely not required for the recruitment of NuRD members to the spindle fiber, as the amount of CHD4 localized to the metaphase spindle is not affected by overexpression of ZNF512B constructs regardless of their ability to bind the NuRD complex. The observed increase in RBBP4 at the mitotic spindle upon overexpression of the I, but not the ΔI, construct may be explained by the fact that RBBP4 is the direct binding partner of ZNF512B via its iNIM. One can speculate that I-dependent higher levels of RBBP4 compared to CHD4 at the spindle may reflect differences in protein expression levels. It is possible that all endogenous CHD4 protein is already bound at the spindle via endogenous ZNF512B, whereas excess of RBBP4 protein, which is normally not associated with the spindle, is additionally recruited through high levels of overexpressed GFP-ZNF512B-I. While these points require further clarification, it is tempting to speculate that most NuRD complex members interact with the mitotic spindle via distinct binding partners. It is therefore likely that they all fulfill separate, non-redundant functions during mitosis.

Spindle binding of ZNF512B is mediated by its mammalian-specific internal N-terminal domain, as demonstrated by experiments with *X. laevis* GFP-ZNF512B. The IN region contains up to 25 copies of a broadly conserved six-residue motif and is predicted to form a three-faced, right-handed β-helix structure. The predicted structure displays a strip of basic residues along each of the three long edges, creating a highly basic surface arranged in a distinct spatial configuration. It is possible that this arrangement facilitates interaction with a partner possessing a complementary, acidic surface. Interestingly, AlphaFold3 predictions of the IN region with <25 repeats did not lead to a disintegration of the β-helix structure, suggesting that the number of repeats is not crucial for its formation, it just determines the number of coils created. Functionally relevant β-helical structures have been identified in several classes of proteins, including pectate lyase [47] and antifreeze proteins [48, 49]. Notably, the folding and assembly of many triple β-helices depend on a registration signal that regulates the correct three-dimensional structure formation [50]. To date it is unknown whether mitosis signals, such as phosphorylation, also regulate the formation of this putative β-helical structure in ZNF512B.

To explore the possibility of direct tubulin binding, we performed IF microscopy experiments with a GFP-ZNF512B_IN_ΔNLS construct. However, no co-localization with microtubules in the cytoplasm was detected. Possibly, ZNF512B does only bind (mitosis-specific) post-translationally modified tubulin [51] or requires an additional binding partner to localize to the mitotic spindle. Given that the spindle apparatus comprises hundreds of different proteins [52, 53], it remains challenging to identify the direct binding partner of ZNF512B. Our previously published lf-qMS analysis of GFP-ZNF512B interactors [21] and our new lf-qMS data of interactors specifically binding ZNF512B’s internal domain, already provide several potential candidates which are involved in chromosome segregation. Future binding studies and targeted depletion of potential candidates could further refine our understanding of ZNF512B’s function in the complex network of proteins at the mitotic spindle.

Since elevated levels of ZNF512B cause a pronounced delay in metaphase exit, while its depletion accelerates cell proliferation, it is tempting to speculate that ZNF512B plays a direct role in the spindle-dependent orchestration of forces required for sister chromosome segregation. Although our limited microscopic analyses did not reveal major defects in spindle formation, cells appeared to arrest in metaphase, suggesting that mechanisms at the metaphase check point may be affected [54]. This could involve alterations in mechanical forces that directly influence microtubule polymerization [55] or depolymerization dynamics [56]. Alternatively, ZNF512B may impact regulatory components of the metaphase-to-anaphase transition, the so-called spindle assembly checkpoint (SAC) [57].

Another notable observation is that loss of ZNF512B (Zfp512b) leads to faster proliferating mESCs, that fail to properly differentiate into beating CMs or NPCs. This differentiation defect likely results from a combination of mitotic abnormalities and transcriptional deregulation, as ZNF512B also functions as a transcriptional repressor [14, 21]. Supporting our findings, a recent preprint from the group of Charles G. Bailey reported that ZNF512B may play a role in neurogenesis, based on RNAi-mediated depletion experiments in NTERA-2 neural cells [58].

ZNF512B is not the first zinc finger protein reported to have multiple functions, including roles as a transcriptional repressor, regulator of differentiation, and spindle microtubule-associated factor. Other examples include Kaiso, a BTB/POZ domain-containing zinc finger repressor that has been implicated in the regulation of cell cycle progression and cell proliferation in cancer [59], and ZNF207 (BuGZ), a C2H2-type zinc finger protein. Different isoforms of ZNF207 are involved in loading SAC proteins to kinetochores [60] and have also been identified as key drivers of human ESC differentiation into ectoderm lineage [61].

In summary, our findings underscore that zinc finger proteins are not exclusively nuclear transcription factors but can also act as regulators of mitotic progression and of stem cell differentiation.

## Supplementary Material

gkag802_Supplemental_Files

## Data Availability

Raw and processed RNA-seq files are deposited in GSE303898. The mass spectrometry proteomics data have been deposited to the ProteomeXchange Consortium via the PRIDE [62] partner repository with the dataset identifier PXD078292.
