# Supplementary material for: ZNF512B associates with mitotic spindles, regulates metaphase exit, and is crucial for stem cell differentiation
Source: Nucleic Acids Res. 2026 Aug 11;54(15):gkag802. doi: 10.1093/nar/gkag802 (PMC13458389; doi:10.1093/nar/gkag802)
Supplement: Supplementary file 1 [file gkag802_supplemental_files.zip › Supplementary Information + Figures.pdf]

Supplementary Information to

ZNF512B associates with mitotic spindles, regulates metaphase exit and is crucial for stem cell differentiation

Lena W. Paasche[1], Nadine Daus[1,+], Johannes J. Groos[1,+], Felix Diegmüller[1], Carlotta Kreienbaum[1], Erekle Kobakhidze[2], Jie Lan[1], Jörg Leers[1], Stefanie Wunderlich[3], Marion Scheibe[4], Falk Butter[4], Annette Borchers[3], Joel P. Mackay[2], Tim Marius Wunderlich[1,*] and Sandra B. Hake[1,*]

[1]Institute for Genetics, Justus Liebig University Giessen, 35392 Giessen, Germany
[2]School of Life and Environmental Sciences, University of Sydney, New South Wales 2006, Australia
[3]Department of Biology, Molecular Embryology, Marburg University, 35043 Marburg, Germany
[4]Institute of Molecular Virology and Cell Biology, Friedrich-Loeffler-Institute, Federal Research Institute for Animal Health, 17493 Greifswald, Germany

[+]Shared authorship
[*]Corresponding authors: Tim Marius Wunderlich and Sandra B. Hake, Institute for Genetics, Justus Liebig University Giessen, Heinrich-Buff-Ring 58–62, 35392 Giessen, Germany, E-Mails: tim.m.wunderlich@gen.bio.uni-giessen.de and sandra.hake@gen.bio.uni-giessen.de, phones: 0049 (0)641 99 35473 (TMW) or 35460 (SBH), FAX: 0049 (0)641 99 35469

Running title: ZNF512B regulates metaphase exit and cell differentiation

Keywords: ZNF512B / mitotic spindle / metaphase exit / stem cell differentiation / NuRD

Supplementary Material and Methods

Antibodies

Antibody	Host	Supplier	Order Number	Application	Dilution
α-CHD4	Mouse	Abcam	ab70469	IF	1:100
α-H3	Rabbit	Abcam	ab1791	IB	1:5,000
α-RBBP4	Rabbit	Abcam	ab79416	IF	1:100
α-alpha-Tubulin	Mouse	Active Motif	39527	IF	1:100
α-ZNF512B	Rabbit	BJ-Diagnostik BioScience GmbH	not applicable	IF	1:100
α-ZNF512B	Rabbit	Atlas Antibodies	HPA006688	IB	1:1,000
anti-Rabbit IgG (H+L), HRP	Goat	Invitrogen	31460	IB	1:20,000
F(ab')2 anti-Rabbit IgG (H+L) Cross-Adsorbed, Alexa Fluor™ 488	Goat	Invitrogen	A-11070	IF	1:200
F(ab')2 anti-Rabbit IgG (H+L) Cross-Adsorbed, Alexa Fluor™ 594	Goat	Invitrogen	A-11072	IF	1:200
F(ab')2 anti-Mouse IgG (H+L) Cross-Adsorbed, Alexa Fluor™ 594	Goat	Invitrogen	A-11020	IF	1:200

Primers and Oligos

Target / Application		Forward (5' to 3')	Reverse (5' to 3')
ZNF512B	Cloning of GFP-ZNF512B_IN	TCATCGTTCGAAGTCCGGATCTATGGACCGACCCCTGC	TAGTAAGCGGCCGCTCACACCTTCCGCTTCTTCTTGGGTGTGGCACGAGGTGCTTT
	Cloning of GFP-ZNF512B_IC	TCATCGTTCGAAGTCCGGATCTGGGAGGAACAGTGGTAAGAAAAAGGG	TAGTAAGCGGCCGCTCAGACGGCTTCCCCGC
	Cloning of GFP-ZNF512B_IN_ΔNLS	TCATCGTTCGAAGTCCGGATCTATGGACCGACCCCTGC	TAGTAAGCGGCCGCTCATGTGGCACGAGGTGCTTT
	Cloning of GFP-ZNF512B_ΔIN	CCCTGCCTGCCTCCAAGCCTGGGAGGAACAGTGGTAAGAAAAGGG	AGGCTTGGAGGCAGGC
	Cloning of *X. laevis* GFP-Znf512b	CTTCGAAGTCCGGATCTGCTCATCCAATATCTGCTGGGT	CGCGGCCGCTCACTATTACTTCACCCTTCGGGATGG
Generation of *Zfp512b* mESC KOs	Amplifying genomic region for left homology arm	GGACTCACCTGCTTGAACCTTG	GAGAGCTTGGTTCTCGGCTTT
	Amplifying genomic region for right homology arm	ATGGAATCACATGGACCACCC	TAGAGGGACAGGAGGCTTACC
	Left homology arm + 12bp pUC18 and mCherry overhangs for Gibson Cloning	GATTACGAATTCGGCAGGTAGGATAGCTCAG	GCCCTTGCTCACCATCCCTATAGTACAGGAGACAGT

	Left homology arm + 12bp pUC18 and Puromycin overhangs for Gibson Cloning	GATTACGAATTCGGCAGGTAGGATAGCTCAG	CTTGTACTCGGTCATCCCTATAGTACAGGAGACAGT
	Right homology arm + 12 bp triple terminator and pUC18 overhangs for Gibson Cloning	GATGAATAAATTTCAGCAAGCCCATCGG	AGTGCCAAGCTTGTTTGGCCAGGCCTGT
	gRNA 1a/b	CACCGGTATGTCCACTGTGGACCAC	AAACGTGGTCCACAGTGGACATACC
	gRNA 1c/d	CACCGGCCCCACTCTGCTTTGACCC	AAACGGGTCAAAGCAGAGTGGGGCC
	gRNA 2a/b	CACCGTGGTGACCGGCCGGCTGACT	AAACAGTCAGCCGGCCGGTCACCAC
	gRNA 2c/d	CACCGCACCATCAGCCGGCCTGTTG	AAACCAACAGGCCGGCTGATGGTGC
	Amplification of pUC18 backbone with homology arm overhangs	GGCCTGGCCAAACAAGCTTGGCACTGGC	ATCCTACCTGCCGAATTCGTAATCATGGTCATAGCT
	Amplification of mCherry with homology arm overhangs	ACTATAGGGATGGTGAGCAAGGGCGAG	TGGGCTTGCTGAAATTTATTCATCCCACATAACTGAAATTTTATACC
	Amplification of puromycin with homology arm overhangs	ACTATAGGGATGACCGAGTACAAGCCCA	TGGGCTTGCTGAAATTTATTCATCCCACATAACTGAAATTTTATACC
Cloning of pIRES-RFP-H2B	Amplification of RFP-H2B	CTACCGGTCGCCACCATGCCAGAGCCAGCGAAG	AGATCTGAGTCCGGATTAGGCGCCGGTGGAG
	Amplification of pIRES	TCCGGACTCAGATCTCGAGC	GGTGGCGACCGGTAGC
Genomic PCR	Verification of integration of 3x terminator + marker genes in *Zfp512b*	CAGGACCCACCAAAGCTAGG	TTGCTGACACCCACTGGTTT
qPCR Primers	*Zfp512b*	AGGAAGTGCGGAAGAAGGTG	TCCGGGCCACCTGGG
	Hprt1	CTGGTGAAAAGGACCTCTCGAAG	CCAGTTTCACTAATGACACAAACG
	Ctnnb1	CGTGTCCTGTGAAGCCCG	CCAACTCCATCAGGTCAGCTTG
	Tgfbr3	CAGACCAATGGCTACTCGGG	GAGCCTGCACCACAATAGAGT
	Dkk3	AAGGCAAGAGGAGCCATGAATGT	TGGTCTCCACAGCACTCACT
	Wnt3a	TGGCTCCTCTCGGATACCTCT	CACAGCCAAGGACCACCAGAT

Supplementary Figure S1

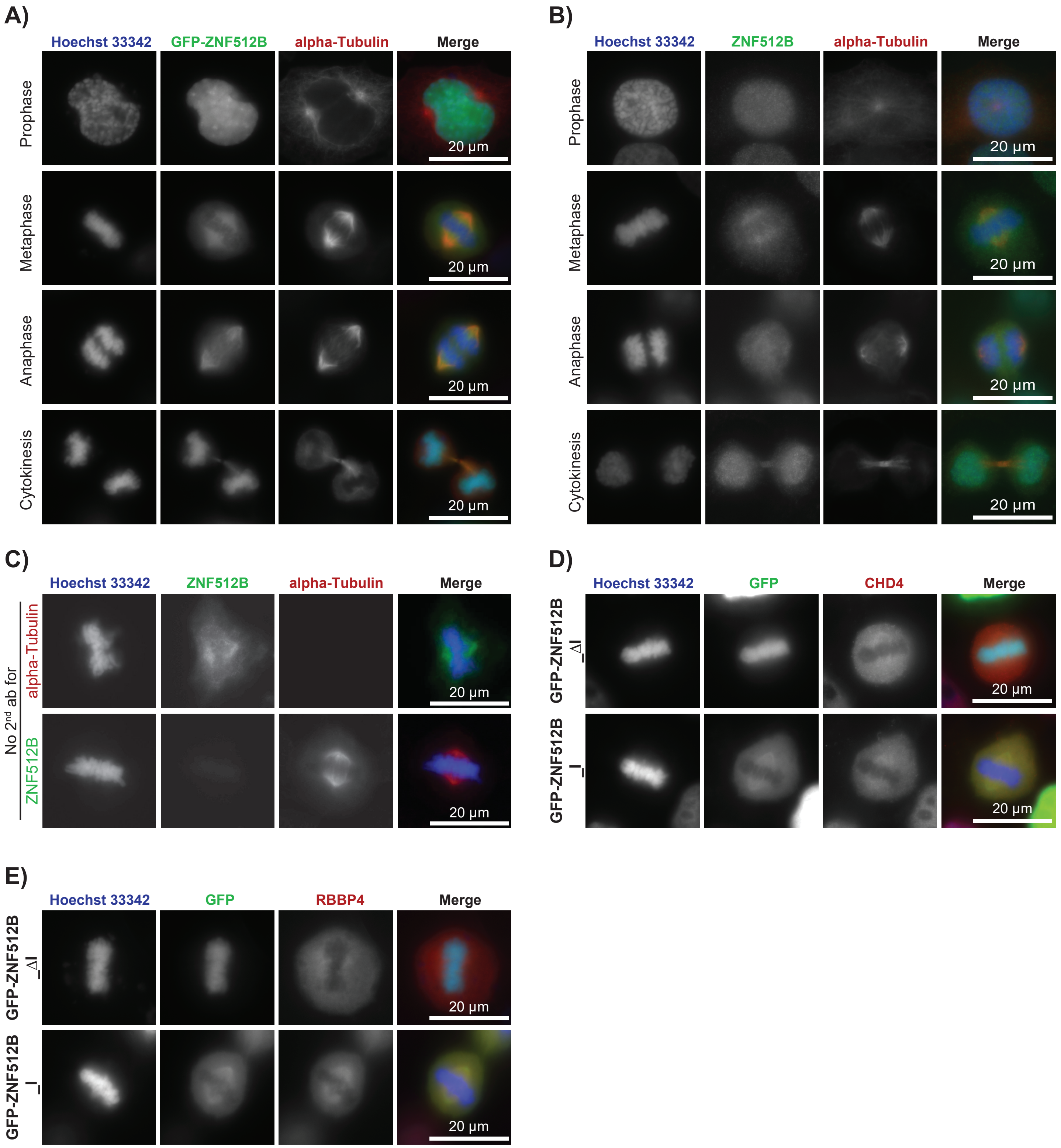

Supplementary Figure S1: RBBP4, in contrast to CHD4, shows increased localization to spindle fibers upon GFP-ZNF512B_I overexpression

(A) Immunofluorescence microscopy pictures of HeLaK cells transfected with GFP-ZNF512B (green). DNA (blue) was visualized by Hoechst 3342 and microtubules/mitotic spindles by anti-alpha-tubulin (red) staining of cells in prophase, metaphase, anaphase and cytokinesis. Scale bar = 20 µm.

(B) Immunofluorescence microscopy pictures of HeLaK cells stained with anti-ZNF512B antibody (green). DNA (blue) was visualized by Hoechst 3342 and microtubules/mitotic spindles by anti-alpha-tubulin (red) staining of cells in prophase, metaphase, anaphase and cytokinesis. Scale bar = 20 µm.

(C) Immunofluorescence microscopy pictures of HeLaK cells stained with anti-ZNF512B (green) and anti-alpha-Tubulin (red) antibodies without addition of respective secondary antibodies as controls. DNA (blue) was visualized by Hoechst 3342. Scale bar = 20 µm.

(D, E) Immunofluorescence microscopy pictures of metaphase HeLaK cells expressing GFP-ZNF512B_I or GFP-ZNF512B_ ΔI (green) and stained with Hoechst 3342 solution (DNA, blue) and **(D)** anti-CHD4 or **(E)** anti-RBBP4 antibodies (red). Scale bar = 20 µm.

Supplemental Figure S2

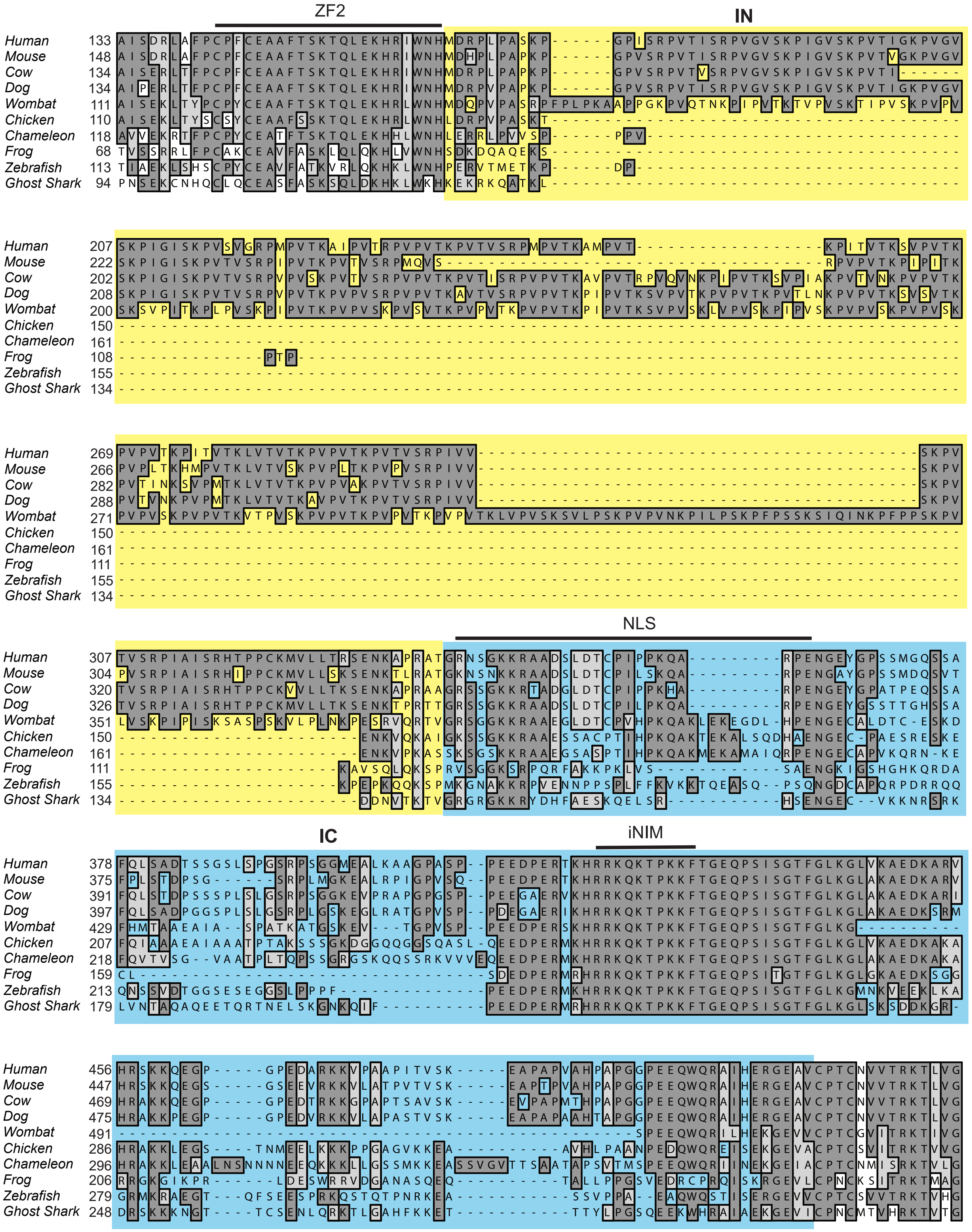

Supplementary Figure S2: Evolutionary conservation of ZNF512B's internal domain.

Protein sequences from human (*Homo sapiens,* Entry Q96KM6), mouse (*Mus musculus,* Entry Q6ZPW1), cow (*Bos taurus,* Entry G3N139), dog (Canis lupus familiaris, Entry A0A8P0NRY1*),* wombat (*Vombatus ursinus*, Entry A0A4X2L7X1), chicken (*Gallus gallus, Entry A0A8V0ZIQ4*), chameleon (*Anolis carolinensis, Entry G1KFN9*), frog (*Xenopus laevis, Entry A0A1L8ELW3*), zebrafish (*Danio rerio, Entry A0A8M2B8T6*) and ghost shark (*Callorhinchus milii*, Entry A0A4W3IEH9) were obtained from UniProt (uniprot.com). Sequence alignment was performed using MacVector software (standard algorithm). Identical amino acids are shaded in dark grey, similar amino acids in light grey and different amino acids in white. Structural features such as zinc finger (ZF), NLS and iNIM regions are indicated by lines. IN is marked by yellow and IC by blue boxes.

Supplementary Figure S3

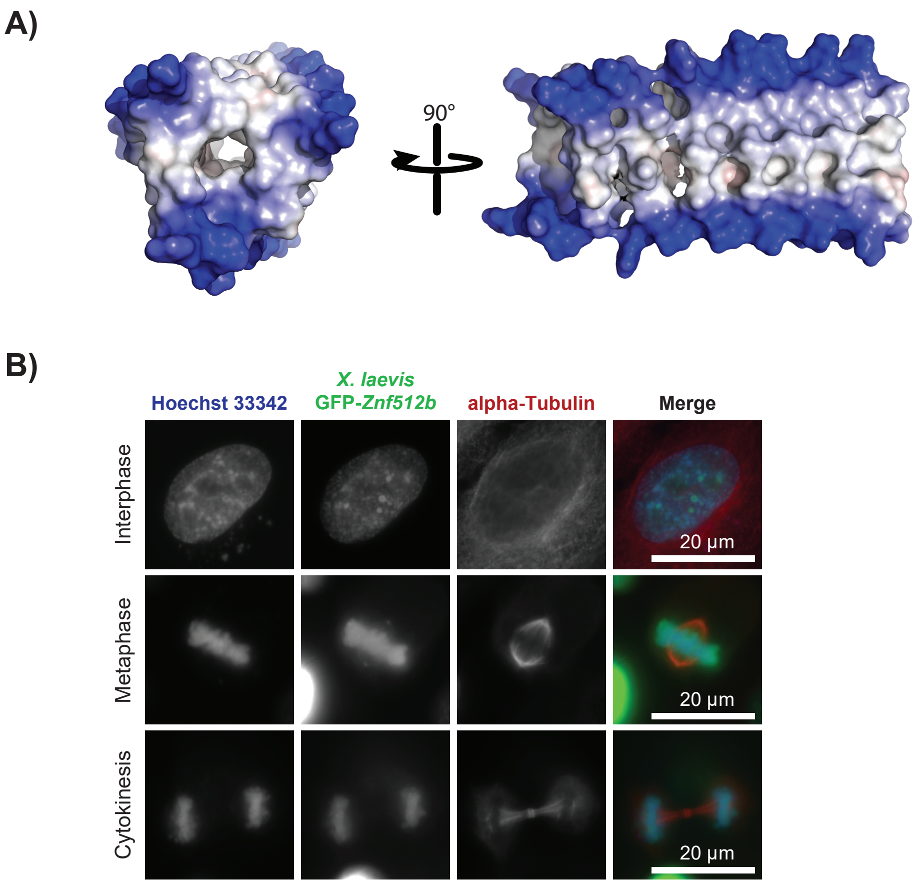

Supplementary Figure S3: Function and structure of ZNF512B's N-terminal internal domain.

(A) Electrostatic surface prediction (calculated in Pymol 3.1) of the AlphaFold2 prediction for human ZNF512B's IN domain. Blue, grey and red indicate positive, neutral and negative electrostatic potential, respectively. Shown are two views rotated 90°.

(B) Immunofluorescence microscopy pictures of HeLaK cells transfected with a GFP-tagged *Xenopus laevis* Znf512b construct (green) in interphase, metaphase or cytokinesis. DNA (blue) was visualized by Hoechst 3342 and microtubules/mitotic spindles by anti-alpha-tubulin (red) staining. Scale bar = 20 µm.

Supplementary Figure S4

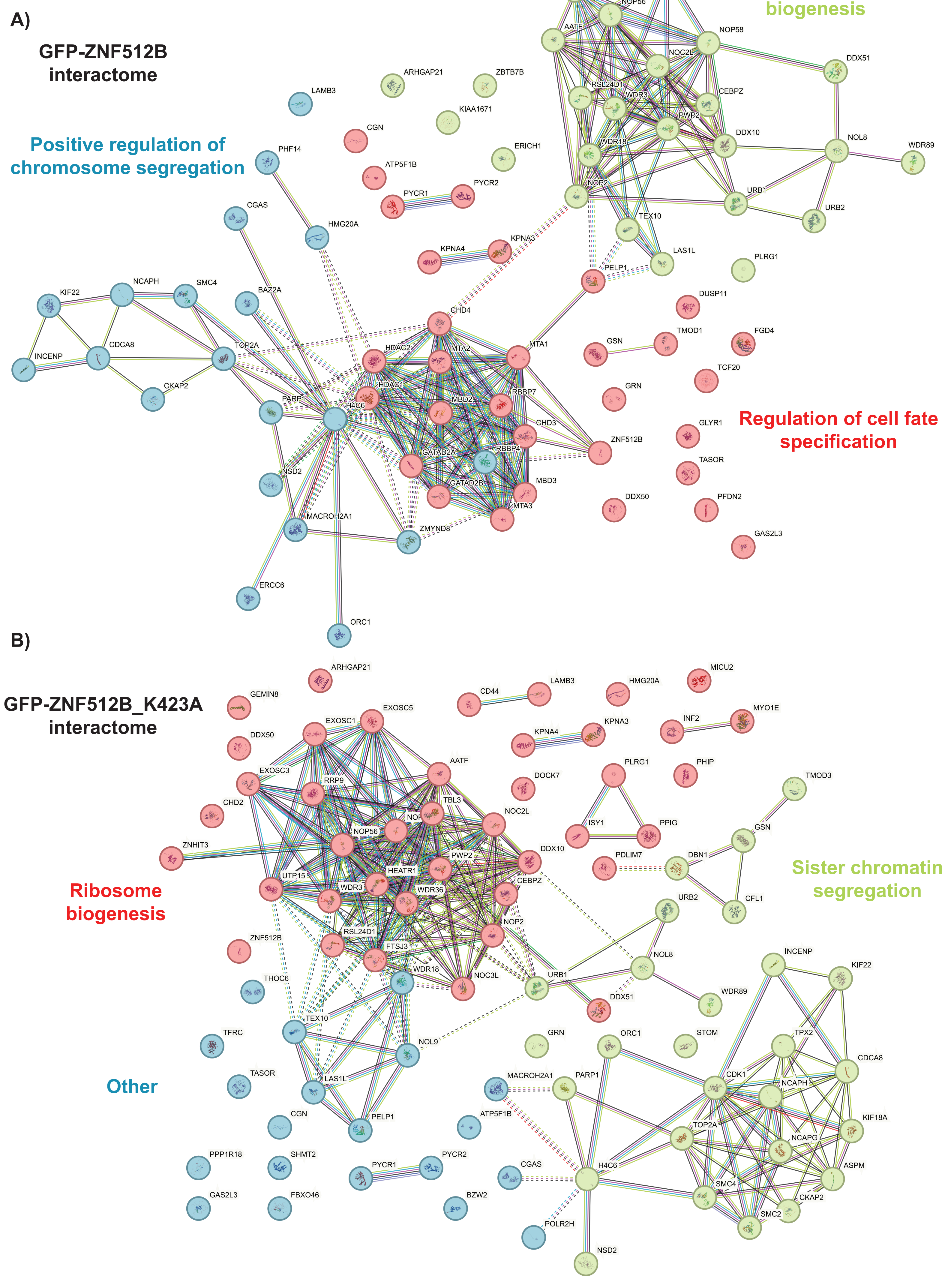

C)

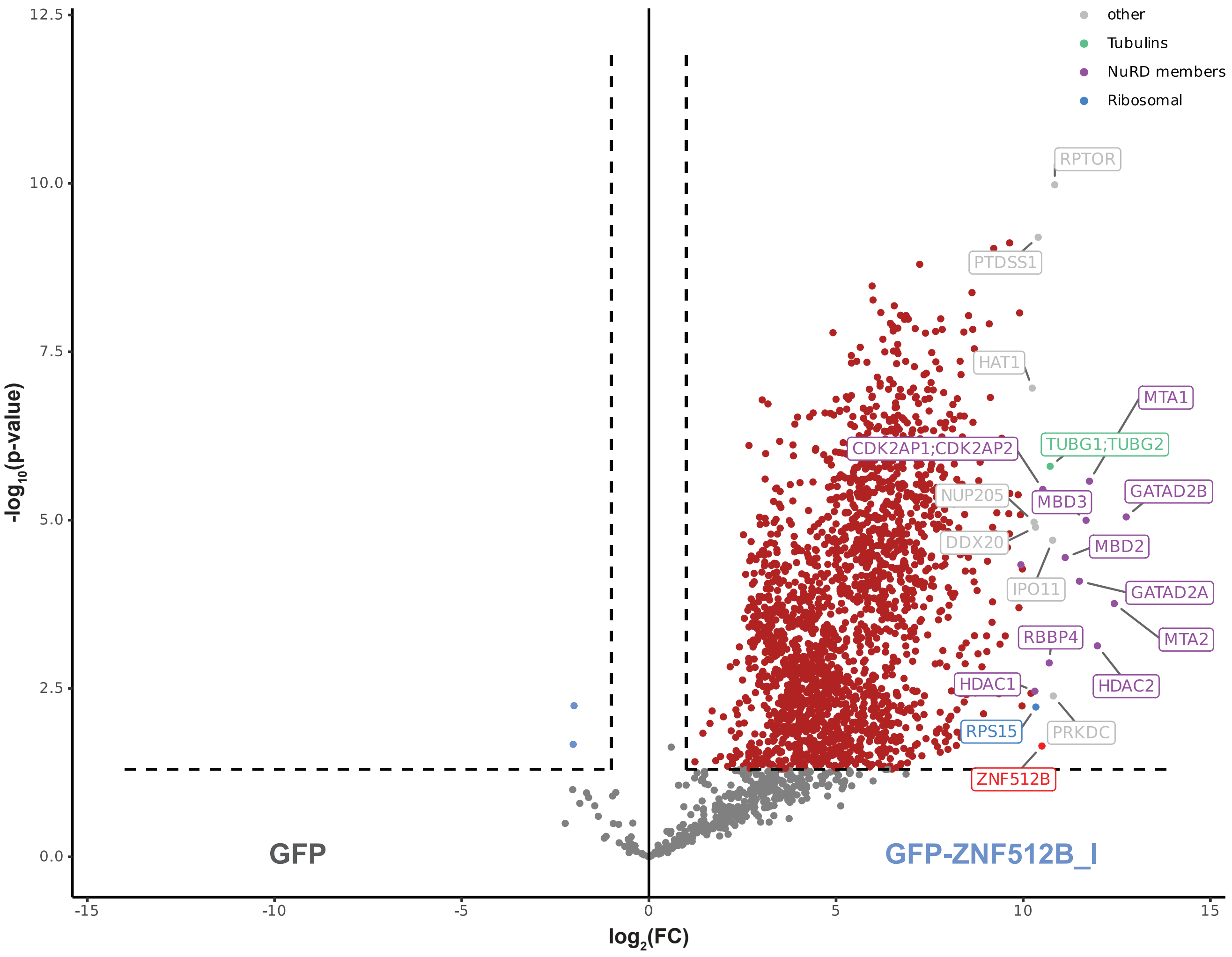
other
Tubulins
NuRD members
Ribosomal
RPTOR
PTDSS1
HAT1
MTA1
TUBG1;TUBG2
CDK2AP1;CDK2AP2
GATAD2B
NUP205
MBD3
DDX20
MBD2
IPO11
GATAD2A
RBBP4
MTA2
HDAC1
HDAC2
RPS15
PRKDC
ZNF512B
GFP
GFP-ZNF512B_I
-log10(p-value)
log2(FC)
12.5
10.0
7.5
5.0
2.5
0.0
-15
-10
-5
0
5
10
15

D)

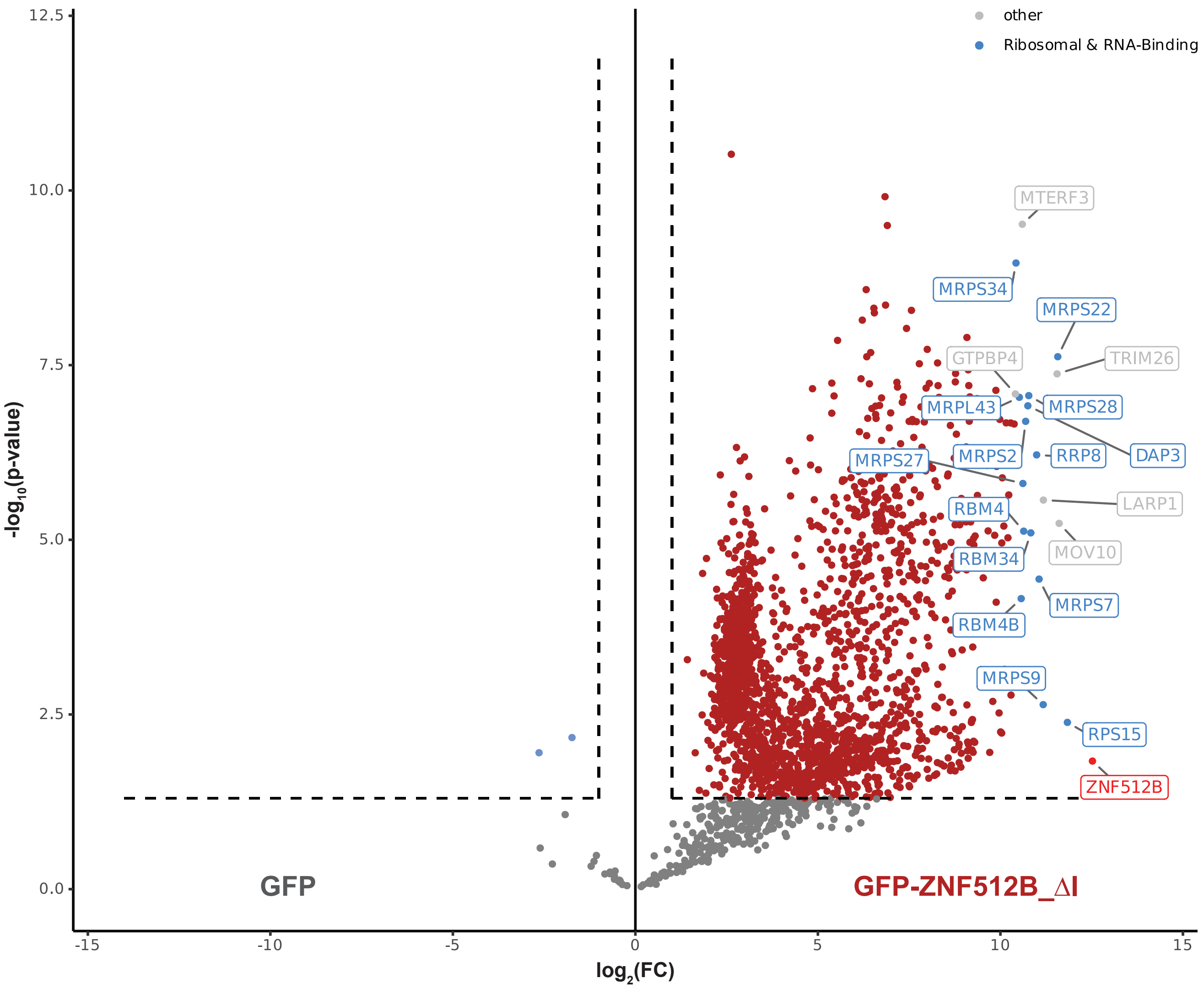
other
Ribosomal & RNA-Binding
MTERF3
MRPS34
MRPS22
GTPBP4
TRIM26
MRPL43
MRPS28
MRPS27
MRPS2
RRP8
DAP3
LARP1
RBM4
MOV10
RBM34
MRPS7
RBM4B
MRPS9
RPS15
ZNF512B
GFP
GFP-ZNF512B_ΔI
-log10(p-value)
log2(FC)
12.5
10.0
7.5
5.0
2.5
0.0
-15
-10
-5
0
5
10
15

Supplementary Figure S4: ZNF512B interacts with proteins involved in chromosome segregation, ribosome biogenesis and regulation of cell fate specification

(A, B) STRING network analysis of proteins interacting with **(A)** GFP-ZNF512B or **(B)** GFP-ZNF512B_K423A identified through label-free quantitative mass spectrometry (lf-qMS) (1). Clustering of protein-protein-interaction (PPI) networks was performed by k-means with PPI enrichment p-value < 10.e-16. Different colors represent different biological processes (GO database).

(**C, D)** Volcano plots of lf-qMS data comparing proteins enriched on **(C)** GFP-ZNF512B_I or **(D)** GFP-ZNF512B_ΔI with those bound to GFP. Top 20 most enriched proteins are labelled in each plot. Green dots mark tubulin proteins, purple dots mark NuRD complex members and blue dots mark proteins involved in ribosome biogenesis or RNA-binding. ZNF512B is marked in red. Dashed lines indicate thresholds for significantly enriched proteins (p-value < 0.05, log2FC > 1).

Supplementary Figure S5

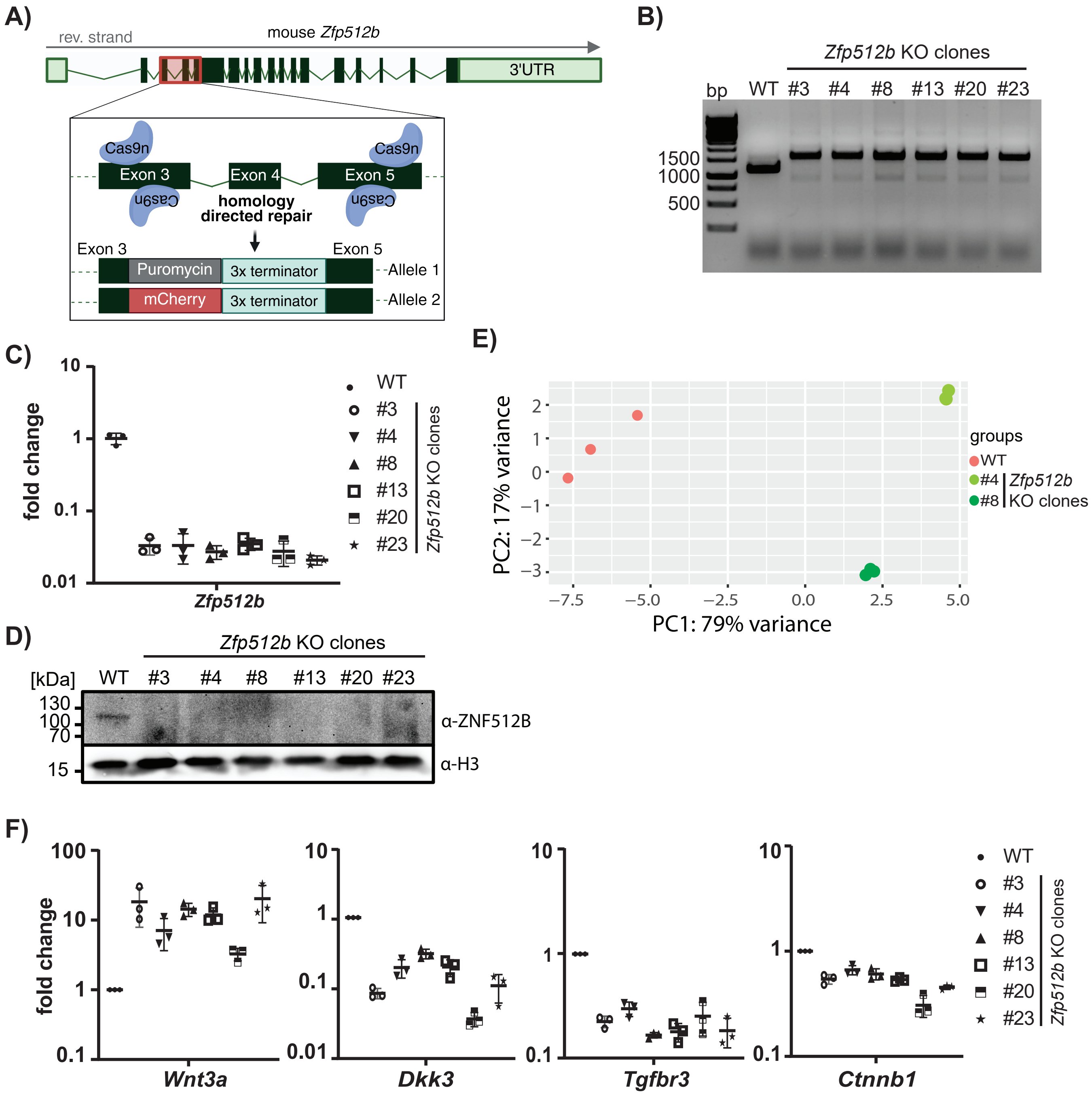

Supplementary Figure S5: Generation and analysis of *Zfp512b* KO mESCs.

(A) Schematic depicting CRISPR/Cas9-based generation of *Zfp512b* KO mESC clones by introducing triple transcriptional terminator sequences together with either puromycin or mCherry selection markers after exon 3 of the murine *Zfp512b* gene.

(B) Agarose gel separation of PCR products after PCR amplification of the murine *Zfp512b* locus using genomic DNA isolated from naïve WT mESCs or six *Zfp512b* KO mESC clones (#3, #4, #8, #13, #20, #23).

(C) RT-qPCR analysis of *Zfp512b* mRNA expression in naïve WT mESCs or six *Zfp512b* KO mESC clones (#3, #4, #8, #13, #20, #23) normalized to HPRT1 expression. Indicated is the fold change in expression compared to WT. Error bars represent the standard deviation of three biological replicates. Significance was calculated in Prism with one-way ANOVA and revealed a significant difference between WT and all mESC *Zfp512b* KO clones of <0.0001.

(D) Immunoblots of nuclear extracts derived from naïve WT mESCs or six *Zfp512b* KO mESC clones (#3, #4, #8, #13, #20, #23) stained with anti-ZNF512B and anti-H3 (loading control) antibodies.

(E) Principle Component Analysis (PCA) of mRNA-seq data from three replicates derived from naïve WT mESCs (red) or *Zfp512b* KO mESC clone #4 (light green) or #8 (dark green).

(F) RT-qPCR analysis of differentially expressed genes *Wnt3a, Tgfbr3, Dkk3* and *Ctnnb1* in naïve WT mESCs or *Zfp512b* KO mESC clones normalized to HPRT1 expression. Indicated is the fold change in expression compared to WT. Error bars represent the standard deviation of three biological replicates. Significance was calculated in Prism with one-way ANOVA and revealed a significant difference between WT and all *Zfp512b* KO mESC clones of <0.0001. Only exception was expression of *Wnt3a,* which showed a significance between WT and clone #3 of <0.05, between WT and clones #4, #8, #13 and #20 of >0.05 and between WT and clone #23 of <0.01.

Supplementary Figure S6

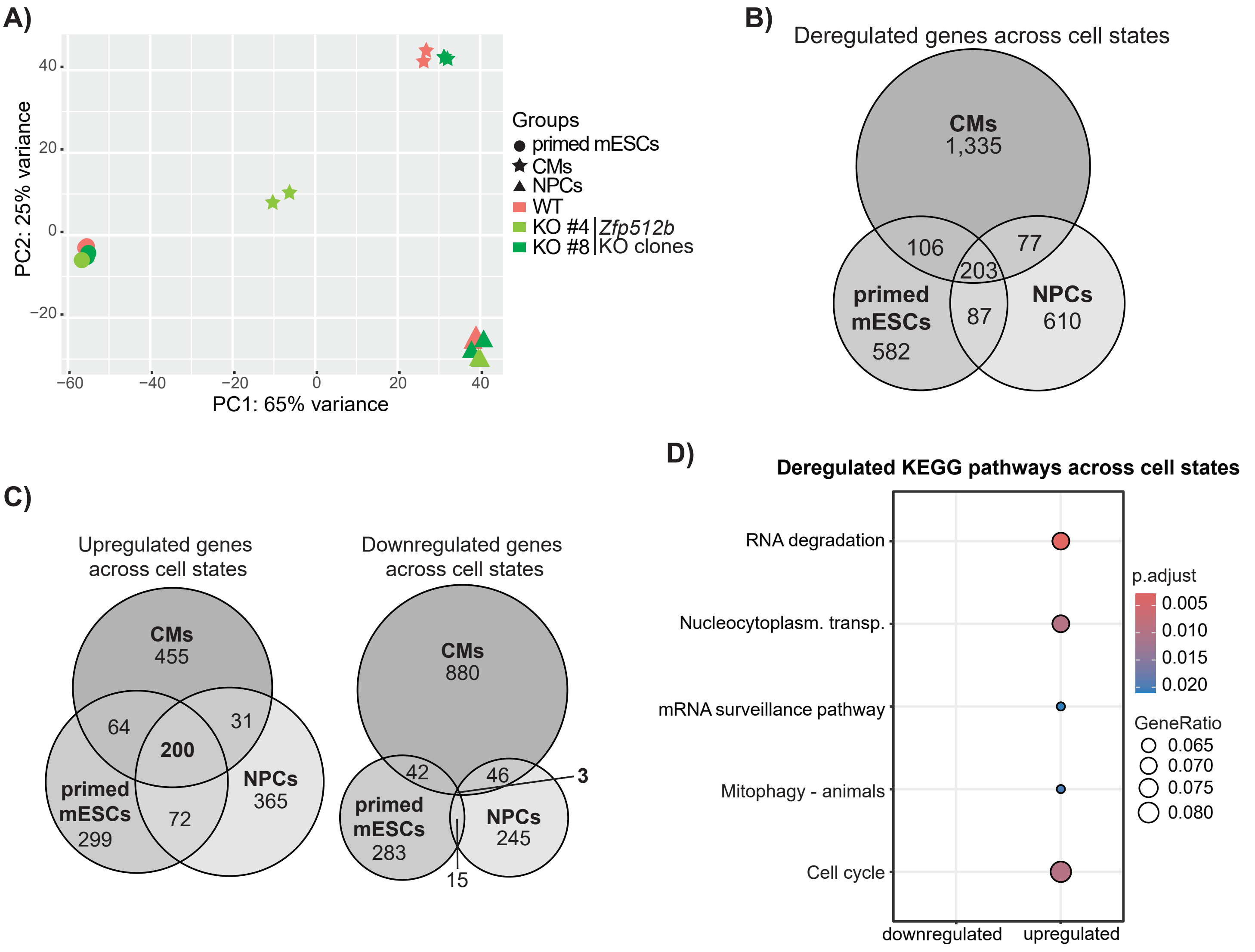

Supplementary Figure S6: ZNF512B depletion results in upregulation of genes associated with the cell cycle across all three differentiation stages.

(A) Principal Component Analysis (PCA) of RNA-seq data from two replicates derived from WT mESCs (red) or *Zfp512b* KO mESC clone #4 (light green) or #8 (dark green) in the primed, NPC or CM stage.

(B) Venn diagram of shared significantly (p-value <0.05) deregulated genes in *Zfp512b* KO clones #4 and #8 compared to WT mESCs across all three stages.

(C) Venn diagrams of shared significantly (p-value <0.05) upregulated (left) or downregulated (right) genes in *Zfp512b* KO clones #4 and #8 compared to WT mESCs in the primed, NPC and CM stage (see **B**).

(D) ORA using the KEGG pathway database of shared significantly (p-value <0.05) deregulated genes in both *Zfp512b* KO clones across all three stages (203 genes shown in **B**).

Supplementary Figure S7

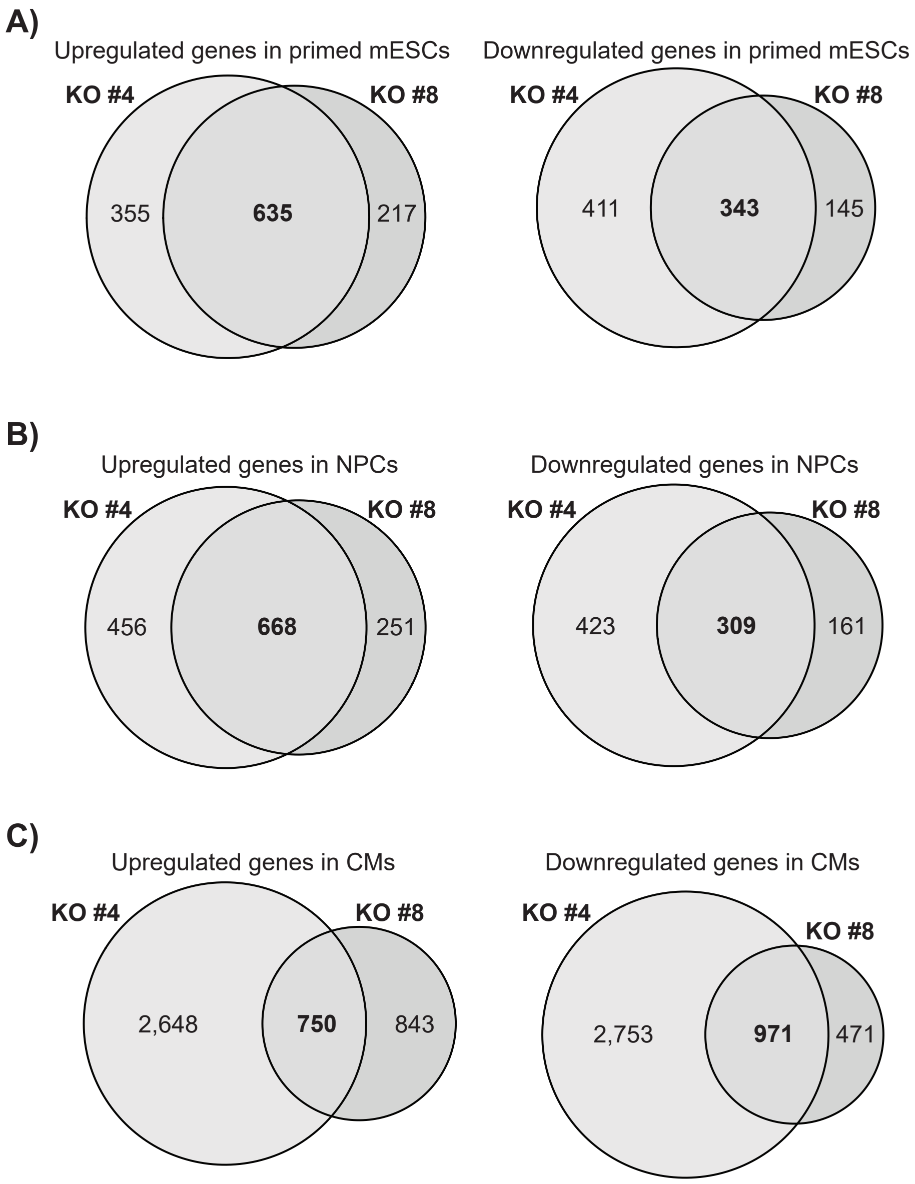

Supplementary Figure S7: ZNF512B depletion results in deregulated gene expression during mESC differentiation into CMs and NPCs.

(A–C) Venn diagrams of significantly (p-value <0.05) upregulated (left) or downregulated (right) genes in *Zfp512b* KO clones #4 and #8 compared to WT cells in the **(A)** primed, **(B)** NPC or **(C)** CM stage.

Movie Legends

Movie 1:

Live-cell 3D reconstruction of a HeLaK cell in metaphase transiently expressing RFP-H2B and GFP-ZNF512B. Twenty z-stacks were acquired with a Spinning-Disc microscope at 0.65 µm intervals and reconstructed using Imaris. Scale bar = 5 µm.

Movies 2–8:

Videos of live-cell imaging from different time points of HeLaK cells stably expressing RFP-H2B (red) transfected with GFP (Movie 2), GFP-ZNF512B (Movie 3), GFP-ZNF512B_K423A (Movie 4), GFP-ZNF512B_ΔI (Movie 5), GFP-ZNF512B_I (Movie 6), GFP-ZNF512B_IN (Movie 7) and GFP-ZNF512B_IC (Movie 8). Cells were monitored over a 36-hour time period, with images acquired every 20 minutes using the CELLCYTE X (Cytena) live-cell imaging system. Movies are shown with 2.5 frames per second. Scale bar = 100 µm.

Supplementary Table Legends

Supplementary Table 1:

RNA-seq data of shared deregulated genes in naïve *Zfp512b* KO clones #4 and #8 compared to naïve WT mESCs.

Supplementary Table 2:

RNA-seq data of shared deregulated genes in *Zfp512b* KO clones #4 and #8 compared to WT cells in primed, NPC and CM stages.

References

1. Wunderlich, T.M., Deshpande, C., Paasche, L.W., Friedrich, T., Diegmuller, F., Haddad, E., Kreienbaum, C., Naseer, H., Stebel, S.E., Daus, N. *et al.* (2024) ZNF512B binds RBBP4 via a variant NuRD interaction motif and aggregates chromatin in a NuRD complex-independent manner. *Nucleic acids research*, **52**, 12831-12849.
